# First molecular evidence of hybridization in endosymbiotic ciliates (Protista, Ciliophora)

**DOI:** 10.3389/fmicb.2022.1067315

**Published:** 2022-12-08

**Authors:** Tomáš Obert, Tengyue Zhang, Ivan Rurik, Peter Vďačný

**Affiliations:** Department of Zoology, Faculty of Natural Sciences, Comenius University in Bratislava, Bratislava, Slovakia

**Keywords:** Central Europe, cytochrome *c* oxidase subunit I, diversification, lumbricid earthworms, nuclear-cytoplasmic discordance, *Plagiotoma aporrectodeae* sp. n., rDNA cistron

## Abstract

Hybridization is an important evolutionary process that can fuel diversification *via* formation of hybrid species or can lead to fusion of previously separated lineages by forming highly diverse species complexes. We provide here the first molecular evidence of hybridization in wild populations of ciliates, a highly diverse group of free-living and symbiotic eukaryotic microbes. The impact of hybridization was studied on the model of *Plagiotoma*, an obligate endosymbiont of the digestive tube of earthworms, using split decomposition analyses and species networks, 2D modeling of the nuclear rRNA molecules and compensatory base change analyses as well as multidimensional morphometrics. Gene flow slowed down and eventually hampered the diversification of *Lumbricus*-dwelling plagiotomids, which collapsed into a single highly variable biological entity, the *P. lumbrici* complex. Disruption of the species boundaries was suggested also by the continuum of morphological variability in the phenotypic space. On the other hand, hybridization conspicuously increased diversity in the nuclear rDNA cistron and somewhat weakened the host structural specificity of the *P. lumbrici* complex, whose members colonize a variety of phylogenetically closely related anecic and epigeic earthworms. By contrast, another recorded species, *P. aporrectodeae* sp. n., showed no signs of introgression, no variability in the rDNA cistron, and very high host specificity. These contrasting eco-evolutionary patterns indicate that hybridization might decrease the alpha-diversity by dissolving species boundaries, weaken the structural host specificity by broadening ecological amplitudes, and increase the nuclear rDNA variability by overcoming concerted evolution within the *P. lumbrici* species complex.

## Introduction

Clusters of morphologically indistinguishable or nearly indistinguishable but genetically well-separated populations of ciliates have been recognized since Sonneborn’s (1938, 1939) first studies on the model organism, the slipper animalcule, *Paramecium aurelia* Ehrenberg, 1838. Such populations were originally called forms or varieties and, later on, they were termed syngens. These were traditionally distinguished from each other by mating reactions with a set of cultured reference strains. Eventually, binomial species names have been assigned to syngens (Sonneborn, 1975; Nanney and McCoy, 1976; Steinbrück and Schlegel, 1983), and their identification is nowadays routinely based on sequences of the mitochondrial barcoding gene coding for cytochrome *c* oxidase subunit I (COI) and/or mating experiments (e.g., Fokin et al., 2004; Barth et al., 2006; Tarcz et al., 2012; Przyboś and Tarcz, 2013, 2016, 2019; Krenek et al., 2015; Potekhin and Mayén-Estrada, 2020; Greczek-Stachura et al., 2021). Soon after the discovery of syngens in other groups of ciliates emerged a question of how capable these “molecular” species are to maintain sharp boundary lines for their gene pools. Inter-specific mating was, for instance, noticed between some species of the *Euplotes crassus-minuta-vannus* group already in the ‘60s of the past century (Nobili, 1964). *Euplotes* hybrids (*crassus* × *vannus*) were, however, usually inviable and episodes of inter-specific mating were apparently magnified under laboratory conditions that were optimized to promote sexual reproduction. It was, therefore, assumed that hybridization in nature may occur only seldom in *Euplotes* (Valbonesi et al., 1988) and possibly also in other ciliate genera. Indeed, obvious discordances between nuclear and mitochondrial data, which are predominantly caused by hybridization, have been up to now not detected in wild populations of free-living and symbiotic ciliates (e.g., Shazib et al., 2019; Rataj and Vďačný, 2020, 2021, 2022; Obert et al., 2021; Zhang and Vďačný, 2021a,b, 2022; Rataj et al., 2022). It was, therefore, a great surprise when we noticed a strong conflicting signal between the nuclear rDNA cistron and the mitochondrial COI phylogenies in the genus *Plagiotoma* Dujardin, 1841 (family Plagiotomidae Bütschli, 1887, class Spirotrichea Bütschli, 1889), which lives in the digestive tube of earthworms.

Besides plagiotomids, the digestive tract of lumbricid earthworms is colonized by three phylogenetically fairly distant ciliate groups: nyctotherids (family Nyctotheridae Amaro, 1972, class Armophorea Lynn, 2004), astomes (subclass Astomatia Schewiakoff, 1896, class Oligohymenophorea de Puytorac et al., 1974), and hysterocinetids (family Hysterocinetidae Diesing, 1866, class Oligohymenophorea). There are, however, huge differences in the known alpha-diversity of these four endosymbiotic ciliate groups. Plagiotomids comprise only a few species (Albaret, 1975; Albaret and Njiné, 1975; Mandal and Nair, 1975) while hysterocinetids, nyctotherids, and astomes are highly diversified accounting for several hundred taxa (for reviews, see de Puytorac, 1954; Raabe, 1972; Albaret, 1975). Such uneven distribution of diversity provokes a question of what are the reasons for this pronounced asymmetry among these four phylogenetically distant ciliate groups. Diversification and distribution of endobiotic ciliates are very likely governed by the geographic range and biology of their hosts (e.g., Irwin et al., 2017; Vďačný, 2018; Rataj and Vďačný, 2020, 2021; Obert et al., 2021; Zhang and Vďačný, 2021a,b, 2022; Rataj et al., 2022). However, host switching and hybridization also have a significant impact on diversification and speciation processes. Hybridization can accelerate diversification *via* formation of hybrid species (Seehausen, 2004; Litsios and Salamin, 2014; Stankowski and Streisfeld, 2015; Sefc et al., 2017; Ebersbach et al., 2020; White et al., 2020), but it can also slow down diversification *via* breakdown of species reproductive barriers (Huxel, 1999; Gómez et al., 2015; Todesco et al., 2016). Disruption of species boundaries can thus lead to the fusion of previously separated lineages and, hence, to a decrease in the alpha-diversity.

Using comprehensive sampling at mesoscale and a combination of nuclear rDNA cistron and mitochondrial COI sequences with morphometric and cell geometric data, we aim to address the following questions:

1. Was the alpha-diversity of plagiotomids in the digestive tract of lumbricid earthworms underestimated? It is expected that the plagiotomid diversity might have been strongly underrated, since only morphological observations were used for species identification in the past (Dujardin, 1841; Cordero, 1928; Pertzewa, 1929; Heidenreich, 1935; de Puytorac and Mauret, 1956; Dworakowska, 1966; Albaret, 1973, 1975). Moreover, all plagiotomids isolated from lumbricid earthworms were identified as a single species, *Plagiotoma lumbrici* (Schrank, 1803) Dujardin, 1841. This further provokes a question of whether this species is broadly distributed, showing a rather weak structural host specificity.
2. Do plagiotomids cluster according to the ecological groups of their lumbricid hosts? To address this question, we investigated three ecological groups of earthworms at mesoscale: (1) epigeic (litter or surface-dwelling) earthworms that form no or only a few burrows and feed on decomposing organic material; (2) anecic (topsoil-dwelling) earthworms that live in permanent, vertical burrows and emerge on the soil surface to feed on dead organic materials mixed with soil; and (3) endogeic (subsoil-dwelling) earthworms that produce a temporary, horizontally oriented burrow systems and feed on soil (Jeffery et al., 2010). If yes, the diversification of plagiotomids is governed by adaptive radiation in association with the ecological group of their earthworm hosts. If not, host switching is common in plagiotomids and their ecological valencies are comparatively broad.
3. What is the extent and effect of hybridization on the diversity of plagiotomids? Does hybridization fuel diversification *via* formation of hybrid species, or does it lead to fusion of previously separated lineages and hence to the formation of a highly diverse species complex?

## Materials and methods

### Material collection and sample processing

Earthworms were collected at 36 localities in southwestern Slovakia, Central Europe (Supplementary Table S1). They were identified using a combination of external morphology (Pižl, 2002) and sequencing of the mitochondrial genes coding for NADH–ubiquinone oxidoreductase chain 1 (ND1) and cytochrome *c* oxidase subunit I (COI). Primers and PCR conditions used for the amplification of ND1 and COI are summarized in Supplementary Tables S2 and S3. Molecular identification of examined earthworms to species is shown in Supplementary Figures S1, S2. Earthworms were processed, dissected, and inspected for the presence of ciliates as described by Obert and Vďačný (2019, 2020a,b).

### Taxonomic methods and morphometric analyses

Living ciliates were manually isolated from the gut content of their earthworm hosts with Pasteur micropipettes and investigated *in vivo* at low (50–400 ×) and high (1,000 ×, oil immersion) magnifications with bright field and differential interference contrast under a Leica DM2500 optical microscope following Foissner (2014). The ciliary pattern and nuclear apparatus were revealed with protargol impregnation (Wilbert, 1975). Capturing of photomicrographs, preparation of illustrations, and measurements were conducted as described elsewhere (Zhang and Vďačný, 2021a,b). Terminology mostly follows Berger (1999, 2006).

Altogether, 21 quantitative features were scored on 52 protargol-impregnated specimens originating from epigeic, anecic, and endogeic lumbricid earthworms (Supplementary Table S4). The morphometric matrix was processed in Python ver. 3.6.6, using the libraries NumPy (Oliphant, 2015) and Pandas (McKinney, 2010). Pairwise similarities of *Plagiotoma* individuals were measured with Euclidean distance. Since this coefficient depends on the scale at which characters are measured, standardization by standard deviation was used to avoid unequal influences on the results (Marhold, 2011). Principal component analysis (PCA) was conducted on the pairwise standardized Euclidean similarity matrix, using the scikit-learn package (Pedregosa et al., 2011). Plotting of the PCA ordination diagram was done with Matplotlib (Hunter, 2007).

The same specimens that had been used for the morphometric multivariate analyses were captured by a Canon EOS 70D camera. Micrographs were imported into Inkscape ver. 0.92^1^ and processed to generate cell outlines. These were statistically analyzed with the package Momocs,^2^ as implemented in the environment R ver. 4.0.3 (R Development Core Team, 2020). First, the number of harmonics was determined, i.e., outlines were decomposed into a sum of trigonometric functions. Frequencies of these functions are integer multiples and hence harmonics of one another. The total cumulative harmonic power of 99.9% was obtained already with 13 harmonics. Consequently, the elliptical Fourier analysis was performed with the number of harmonics set to 13. Fourier coefficients were normalized and used for PCA and hierarchical clustering. Finally, multivariate analysis of variance (MANOVA) was performed on the PCA objects and served for testing of difference between body shapes of plagiotomids isolated from different ecological groups of lumbricid earthworms.

### Molecular methods

Single cells were placed in 180 μl of cell lysis buffer (Promega, Fitchburg, Wisconsin, United States), and their genomic DNA was isolated using the ReliaPrep^™^ Blood gDNA Miniprep System (Promega, Fitchburg, Wisconsin, United States). Three nuclear genes (18S, 5.8S, and 28S), their internal transcribed spacers (ITS1 and ITS2), and the barcoding mitochondrial COI gene were PCR amplified. Primers and PCR conditions are provided in Supplementary Tables S2 and S3. PCR reactions were carried out with the GoTaq^®^ Long PCR Master Mix (Promega, Fitchburg, Wisconsin, United States), following the protocol described in our previous studies (Obert and Vďačný, 2019, 2020a,b; Obert et al., 2021). Bidirectional Sanger sequencing was conducted in Macrogen Europe B.V. (Amsterdam, Netherlands) on an ABI 3730 automatic sequencer.

### Alignment procedures and predicting secondary structures

Nuclear rRNA genes and their spacers were aligned according to the primary and the predicted secondary structures using the package 4SALE ver. 1.7.1 (Seibel et al., 2006). Secondary structures of 18S, 5.8S, and 28S rRNA molecules were constructed with R2DT (Sweeney et al., 2021), considering the models proposed for *Tetrahymena thermophila* (Lee and Gutell, 2012). Since helix 25 of the 28S rRNA molecule is hypervariable, its structure was adjusted with the free-energy minimization approach on the Mfold web server ver. 3.0 (http://www.unafold.org/) (Zuker, 2003). Modeling of ITS2 followed Obert and Vďačný (2020b). Secondary structures were plotted either in 4SALE (Seibel et al., 2006) or TRAVeLer (Elias and Hoksza, 2017). The helix number system of rRNA molecules was according to Lee and Gutell (2012) and Petrov et al. (2014). The mitochondrial protein-coding COI sequences were aligned with MEGA X (Kumar et al., 2018), using the protozoan mitochondrial genetic code (translation table 4) and the Muscle codon algorithm.

### Gene trees

Nuclear rDNA and mitochondrial COI trees were constructed in a neighbor-joining (NJ), maximum likelihood (ML) and Bayesian framework. NJ analyses were conducted in MEGA X and included the maximum composite likelihood method, gamma-distributed rates among sites, a heterogeneous pattern among lineages, a pairwise deletion option to exclude alignment gaps, and 5,000 bootstrap pseudo-replicates. ML analyses were carried out in IQ-TREE ver. 1.6.10 (Nguyen et al., 2015), while Bayesian inferences (BI) were performed in MrBayes ver. 3.2.7 (Ronquist et al., 2012). Settings in ML analyses were as follows: (1) the best substitution model, as selected under the Bayesian information criterion, was assigned to each partition, (2) the edge-unlinked partition model that accounts for heterotachy (rate variation across sites and lineages) and allows each partition to have its own set of branch lengths, (3) thousand ultrafast bootstrap pseudo-replicates, and (4) the bnni algorithm to reduce overestimating bootstrap support. All other parameters were left default. Settings of Bayesian analyses were as follows: (1) prior parameters of evolutionary models as estimated with IQ-TREE, (2) model parameters were unlinked across partitions, (3) one million Markov chain Monte Carlo (MCMC) simulations, (4) a sampling frequency of trees and parameters at one hundred, and (5) a relative burn-in fraction of 25%. Convergence of MCMC analyses was confirmed with the in-built diagnostics of the program MrBayes. Trees were visualized in FigTree ver. 1.4.3.^3^

### Species trees and species delimitation

Species trees were calculated under the Bayesian multispecies coalescent model, using *BEAST ver. 1.8.3 (Heled and Drummond, 2010). Input files were prepared in BEAUti ver. 1.8.3 with the following settings: (1) best evolutionary substitution models as selected by IQ-TREE for each partition; (2) four categories for substitution rate heterogeneity; (3) uncorrelated lognormal clock; (4) ploidy scalars at 1.0 for the mitochondrial partition and 2.0 for the nuclear partition; (5) the Yule process model for the species tree prior; (6) piecewise constant population size, and (7) 100 million generations and a sampling frequency of 1,000 in Markov Chain Monte Carlo analyses. The convergence to stationary distribution (effective sample size > 200 for all parameters) was checked in Tracer ver. 1.6 (Rambaut et al., 2018). The maximum clade credibility tree was summarised in TreeAnnotator ver. 1.8.1 after discarding the first 10% of sampled trees.

Bayesian species delimitation was conducted in BP&P ver. 2.2 (Yang, 2015), with the same datasets as used in the construction of species trees. The maximum clade credibility tree obtained with *BEAST served as a guide tree for species delimitation. Each species delimitation model was assigned equal prior probability. Prior parameters for the ancestral population size θ and root age *τ* were estimated by running A00 analyses. The rjMCMC analyses were run for 100,000 generations with a sampling frequency of 2 and a burn-in of 10,000. A large fine-tuning parameter (*ε* = 15) was used to guarantee a good mixing in the reversible jump algorithm. All analyses were conducted twice to confirm consistency between runs.

### Neighbor-net analyses and species networks

To visualize conflict in the concatenated nuclear rDNA cistron and mitochondrial COI dataset, a neighbor-net analysis was undertaken with uncorrected distances in SplitsTree ver. 4 (Huson, 1998; Huson and Bryant, 2006). A species network was constructed in PhyloNet ver. 3.6.1, using the maximum pseudo-likelihood framework (Than et al., 2008; Wen et al., 2018). The network was computed from the nuclear rDNA and mitochondrial COI trees, allowing for a maximum of zero and ten reticulation nodes. Zero reticulations represented a null model corresponding to a species tree, while a maximum of 10 reticulations served to test how many reticulations could be present in the resulting phylogenetic networks. If there were exactly 10 reticulation nodes, another round of analyses with an increased number of reticulations would be needed. However, phylogenetic networks with a maximum of eight reticulations were recovered (see the ‘Results’ section) and hence no further analyses were needed. Each analysis was performed with ten runs and default settings, generating five optimal networks. The species networks were visualized with Dendroscope ver. 2.7.4 (Huson and Scornavacca, 2012).

### Detection of introgressed sequences

To test whether hybridization could be the source of gene tree incongruence, the program JML ver. 1.3.1 was employed (Joly, 2012). This software uses a posterior distribution of species trees, population sizes, and branch lengths to simulate replicate sequence datasets under the coalescence with no migration. The minimum pairwise sequence distance between sequences of two species is evaluated on the simulated datasets and compared to the one estimated from the original data (i.e., from the rDNA cistron and COI datasets). JML analyses were run over 10,000 species trees sampled from the posterior distribution of *BEAST analyses. Settings were as follows: (1) relative mutation rate as estimated from the log file generated during the *BEAST analyses; (2) heredity scalars at 1.0 for the mitochondrial partition and 2.0 for the nuclear partition; and (3) best substitution models as selected under the Bayesian information criterion in IQ-TREE. All pairwise sequence distances with value of *p* < 0.05 were recorded as potential cases of hybridization.

## Results

### Diversity and distribution of plagiotomids at mesoscale

**Figure 1:**
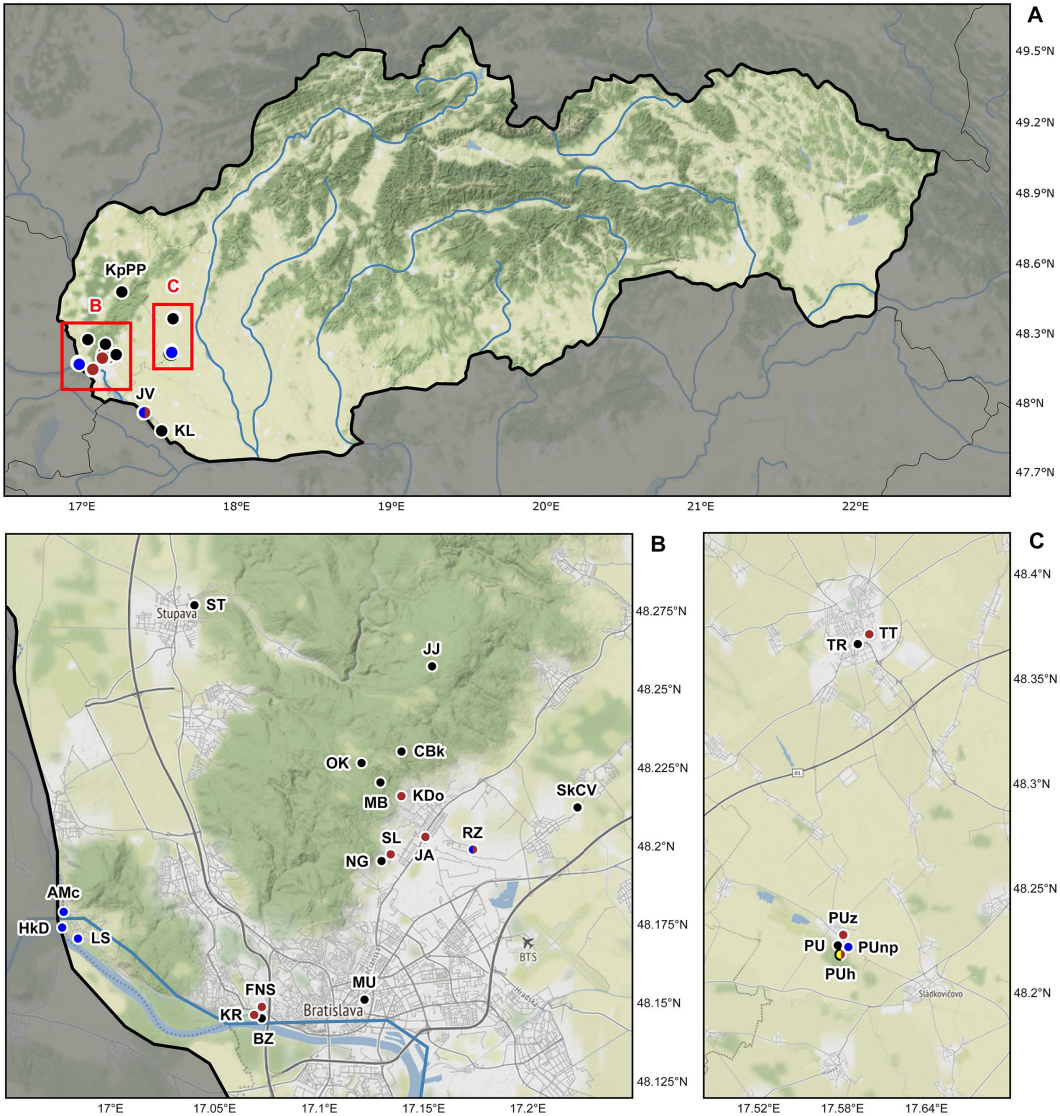
Sampling sites of lumbricid earthworms examined for the presence of plagiotomids in Slovakia, Central Europe. **(A)** Map of Slovakia. Details of two studied areas B and C (marked by red rectangles) are depicted below the map of Slovakia. **(B)** Detail of the Bratislava district showing the localization of 18 sampling sites. **(C)** Detail of the Trnava and Galanta districts showing the localization of six sampling sites. Black dots denote localities where plagiotomids were not recorded, while brown dots denote localities where plagiotomids were isolated from anecic earthworms, yellow dots denote those from epigeic earthworms, and blue dots those from endogeic earthworms. For locality codes, see Supplementary Table S1. Map tiles by ©Stamen Design, under a Creative Commons Attribution (CC BY 3.0) license. Data by OpenStreetMap, under ODbL.

The alpha-diversity of plagiotomids inhabiting the digestive tube of earthworms was studied at mesoscale, more specifically, at 36 localities in southwestern Slovakia, Central Europe (Figure 1). Altogether 880 specimens belonging to 19 earthworm species from three ecological (anecic, epigeic, and endogeic) groups were examined for the presence of plagiotomids (Supplementary Table S1). Only a single earthworm species from each ecological group was, however, colonized by plagiotomids. Namely, the anecic *Lumbricus terrestris* Linnaeus, 1758, the epigeic congener *L. rubellus* Hoffmeister, 1843, and the endogeic *Aporrectodea tuberculata* (Eisen, 1874).

According to the present phylogenetic analyses, as many as nine distinct mitochondrial lineages were recognized within the isolated plagiotomids. One lineage represents a new homogenous and molecularly well-delimited species, *P. aporrectodeae* sp. n., while the remaining eight lineages belong to the highly heterogenous *P. lumbrici* complex (see below). The former species occurred exclusively in the endogeic earthworm *A. tuberculata* and was detected at six out of the seven sites where this endogeic earthworm was sampled (Figure 1; Supplementary Table S1). *Plagiotoma aporrectodeae* sp. n. was never recorded in any *Lumbricus* species even when it co-occurred with *A. tuberculata* (Table 1).

**Table 1:** Distribution of nine mitochondrial lineages of plagiotomids in three earthworm species examined during the course of this study.

Taxon	Lineage	Locality														Host earthworm		
		AMc	HkD	KDo	FNS	JA	JV	KR	LS	RZ	SL	TT	PUh	PUp	PUz	AT	LT	LR
*P. aporrectodeae*		+	+				+		+	+				+		+		
*P. lumbrici* complex	Alpha												+				+	
	Beta												+					+
	Gamma			+								+					+	
	Delta				+		+						+				+	+
	Eta					+				+			+		+		+	
	Theta					+	+	+		+	+	+	+				+	
	Zeta												+					+
	Epsilon												+					+

For locality codes, see Supplementary Table S1. Plus sign (+) designates the presence of a particular lineage. Host earthworms: AT, *Aporrectodea tuberculata*; LT, *Lumbricus terrestris*; LR, *Lumbricus rubellus*.

For the sake of simplicity, mitochondrial lineages of the *P. lumbrici* complex were labeled by the first eight letters of the Greek alphabet (i.e., from alpha to epsilon). Lineages alpha, gamma, eta, and theta were found at nine localities but solely in *L. terrestris*; lineages beta, zeta, and epsilon were isolated only from *L. rubellus* collected just at a single locality; and lineage delta was detected in both *Lumbricus* species sampled at three localities. Theta is apparently the most widespread lineage, as it was recorded at seven out of the 10 sites where members of the *P. lumbrici* complex occurred. Just a single lineage of the *P. lumbrici* complex was found at six localities, while two lineages co-occurred at four localities. Interestingly, at a single spot (PUh), where both *Lumbricus* species carried plagiotomids, as many as seven lineages of the *P. lumbrici* complex were detected (Table 1).

To summarize, there is a correlation between the plagiotomid mitochondrial lineage and the earthworm species. *Plagiotoma aporrectodeae* sp. n. is present only in *A. tuberculata*, lineages alpha, gamma, eta, and theta exclusively in *L. terrestris*, and lineages beta, zeta, and epsilon solely in *L. rubellus*. On the other hand, there is no spatial relationship in the distribution of the plagiotomid lineages, causing the distribution of plagiotomid lineages to be mosaic-like.

### Phylogenetic relationships among plagiotomids

**Figure 2:**
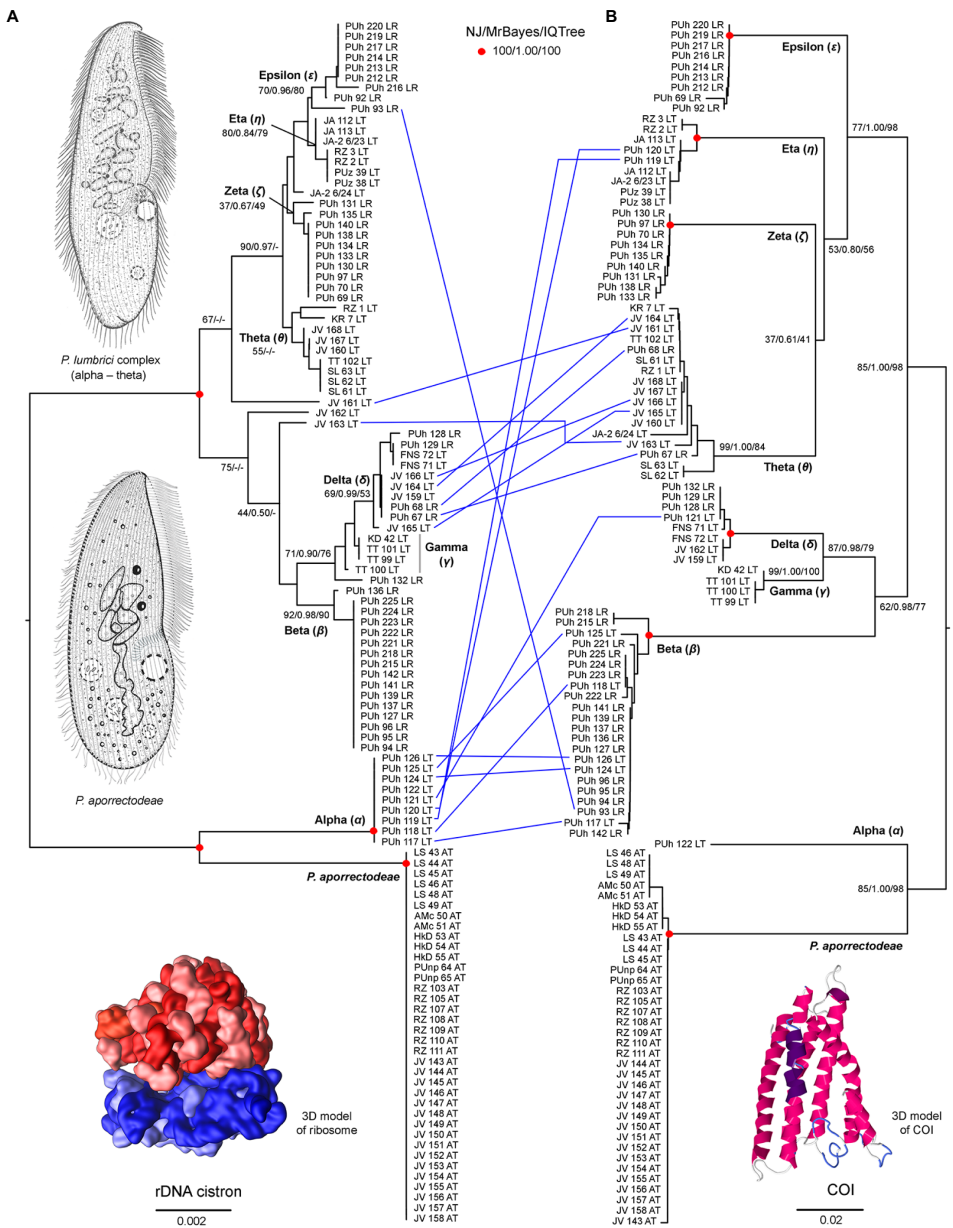
Tanglegram showing congruent and conflicting (blue lines) positions of plagiotomids in the rDNA cistron **(A)** and COI **(B)** trees. Bootstrap values for neighbor-joining (NJ) conducted in MEGA X and for maximum likelihood conducted in IQ-TREE as well as posterior probabilities for Bayesian inferences conducted in MrBayes are listed at corresponding nodes of the NJ trees. According to the present phylogenetic analyses, as many as nine distinct mitochondrial lineages were recognized within the isolated plagiotomids. One lineage represents a new homogenous and molecularly well-delimited species, *P. aporrectodeae* sp. n., while the remaining eight lineages belong to the highly heterogenous *P. lumbrici* complex (labeled by the first eight letters of the Greek alphabet from alpha to epsilon). Conflicting positions of hybrid specimens in the rDNA cistron and COI gene trees are marked by blue lines. For specimen codes and further details, see Supplementary Table S1. Scale bars denote the fraction of substitutions per site. The 3D model of the ribosome is available under the CC BY-SA 3.0 license (Author: Wossman), while the 3D model of COI was constructed using Jmol ver. 14.31.57.

**Figure 3:**
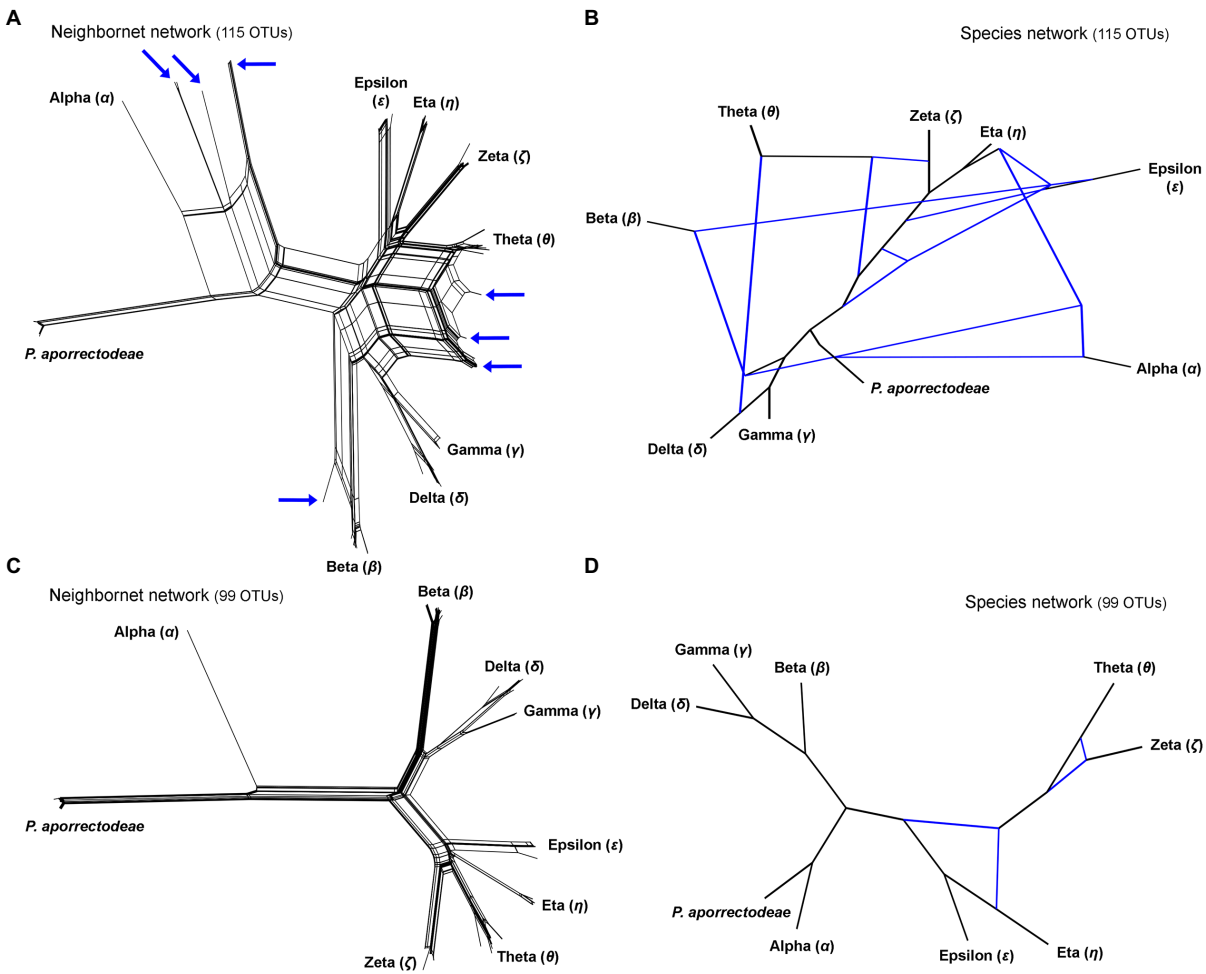
Split decomposition analyses **(A,C)** conducted in SplitsTree and species networks constructed in a pseudo-likelihood framework in PhyloNet **(B,D)**. **(A,C)** Neighbor-net networks constructed from the 115 and 99 specimens datasets. Specimens with conflicting positions in gene trees (see Figure 2) are marked by blue arrows. After their exclusion, the network becomes tree-like as shown in **(C)**. **(B,D)** Species networks constructed from the rDNA cistron and COI trees based on the 115 and 99 specimens datasets. Specimens with conflicting positions in gene trees were excluded from the 99 OTUs dataset.

Nuclear 18S rDNA (GenBank accession numbers OP538845–OP538959), ITS1-5.8S-ITS2 region and the first two domains of 28S rDNA (OP538960–OP539074) as well as the mitochondrial COI (OP562267–OP562381) sequences were obtained from 115 *Plagiotoma* specimens, which had been isolated from three earthworm species originating from 14 localities (Supplementary Table S5). Tree-building and network analyses of the nuclear rDNA cistron and the mitochondrial barcoding COI gene of all 115 *Plagiotoma* specimens revealed phylogenies with rather low resolution and multiple topological discrepancies (Figures 2A,B, 3A). Strong statistical supports and no conflicting signals were detected only in case of *P. aporrectodeae* sp. n. All specimens of this species shared identical rDNA cistron sequences and their COI variability ranged only from 0.00 to 0.58%. By contrast, much higher variability was detected within the *P. lumbrici* complex both in the rDNA cistron (0.00–1.88%, on average 0.73%) and in the COI gene (0.00–11.31%, on average 7.09%). The variability within the three rDNA regions was as follows: 0.00–1.86% (0.60% on average) in the 18S rRNA gene, 0.00–1.72% (0.89% on average) in the ITS1-5.8S-ITS2 region, and 0.00–2.31% (0.87% on average) in the first two barcoding domains of the 28S rRNA gene. Eight distinct mitochondrial lineages were robustly delimited within this species complex by all three tree-building methods (Figure 2B). Inter-lineage distances within the *P. lumbrici* complex ranged from 4.27 to 10.29% in COI sequences. The existence of these eight lineages was, however, either left statistically unsupported or they were paraphyletic in the nuclear rDNA cistron tree (Figure 2A).

**Figure 4:**
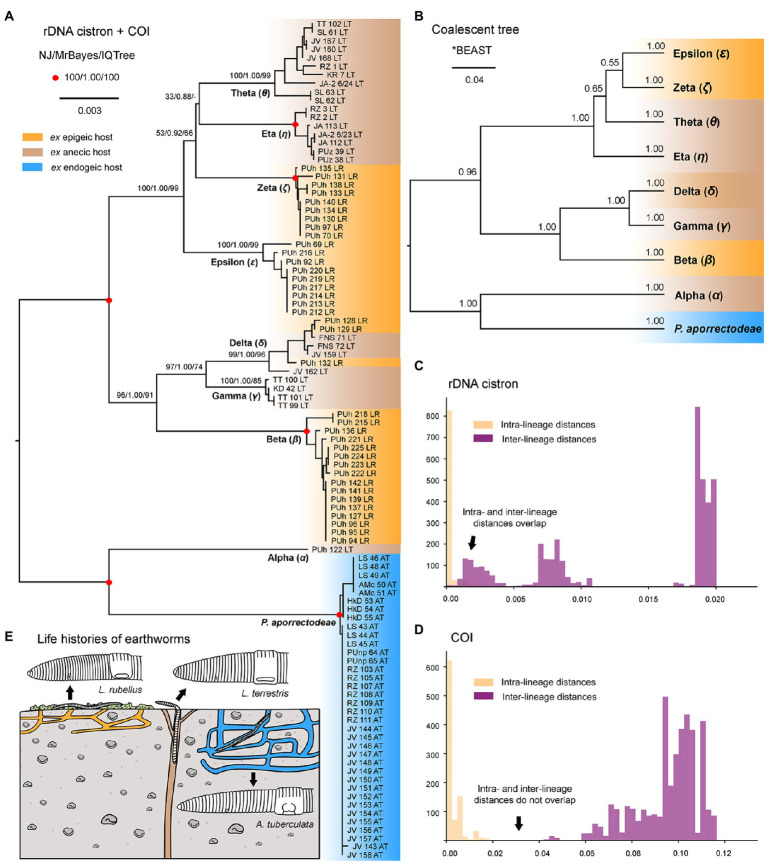
Phylogenetic relationships among plagiotomids isolated from three ecological groups of lumbricid earthworms. **(A)** Phylogenetic tree based on rDNA cistron and COI sequences of 99 specimens. Bootstrap values for neighbor-joining (NJ) conducted in MEGA X and for maximum likelihood conducted in IQ-TREE as well as posterior probabilities for Bayesian inferences conducted in MrBayes are listed at corresponding nodes of the NJ tree. Note that after exclusion of just 16 hybrid specimens, the multi-gene trees became very well resolved and statistically supported. **(B)** Coalescent species tree based on rDNA cistron and COI sequences of 99 specimens. Posterior probabilities of clades are provided along internal branches, while posterior probabilities for the presence of individual species (lineages) are provided above the terminal branches. Scale bars denote the fraction of substitutions per site. **(C, D)** Histograms showing intra- and inter-lineage *p*-distances of rDNA cistron and COI gene sequences of 99 specimens. Individual *Plagiotoma* lineages could not be unambiguously delimited by barcoding analyses of the rDNA cistron *per se*, as intra- and inter-lineage distances partially overlapped. On the other hand, a very distinct barcoding gap was revealed in the COI dataset, whereas the maximum intra-lineage distance was 2.33% while the minimum inter-lineage distance was 4.27%. **(E)** Earthworm life strategies. Epigeic earthworms form no or only a few burrows and feed on decomposing organic material, anecic earthworms live in permanent, vertical burrows and emerge on the soil surface to feed on dead organic materials mixed with soil, and endogeic earthworms produce a temporary, horizontally oriented burrow systems and feed on soil. Colors denote the individual ecological group of earthworms.

The split decomposition diagram inferred from the concatenated rDNA cistron + COI dataset (115 specimens and 3,710 characters) by the neighbor-net algorithm displayed multiple reticulations across the eight lineages of the *P. lumbrici* complex. Individuals causing conflicting signals are placed close to the ‘core’ of lineages alpha, beta, and theta, and they are marked by blue arrows in Figure 3A. Conflicting positions of these specimens in the rDNA cistron and COI gene trees are marked by blue lines in Figures 2A,B. After exclusion of these specimens from the split decomposition analyses, the neighbor-net diagram became tree-like (Figure 3C), resembling the branching pattern suggested by the neighbor-joining rDNA cistron tree (Figure 2A). Gene trees built from the concatenated reduced dataset (99 specimens and 3,710 characters) were very well resolved and statistically supported (Figure 4A). Each lineage within the *P. lumbrici* complex received very high or full support. Lineage alpha grouped with *P. aporrectodeae* sp. n. with very strong support (100% NJ, 1.00 BI, 100% ML). Lineages beta, gamma, and delta formed a monophylum (96% NJ, 1.00 BI, 91% ML). Likewise, lineages eta, theta, zeta, and epsilon clustered together (100% NJ, 1.00 BI, 99% ML) but their interrelationships remain poorly resolved. A very similar picture was obtained also by coalescence-based analyses conducted in *BEAST (Figure 4B). Each plagiotomid lineage received a posterior probability of 1.00 in Bayesian species delimitation analyses conducted in BP&P. Individual lineages could not be, however, unambiguously delimited by barcoding analyses of the rDNA cistron *per se*, as intra- and inter-lineage distances partially overlapped (Figure 4C). On the other hand, a very distinct barcoding gap was revealed in the COI dataset. More specifically, the maximum intra-lineage distance was 2.33% while the minimum inter-lineage distance was 4.27% (Figure 4D).

### Detection of discordant phylogenetic signals within the *Plagiotoma lumbrici* complex

Split decomposition (Figure 3A) and species network (Figure 3B) analyses of the 115 specimens dataset revealed the presence of non-historical signals, whose source might be incomplete lineage sorting, recombination, and/or hybridization. The exclusion of just 16 “problematic” specimens almost completely removed parallel splits from the neighbor-net diagram (see above and Figure 3C) and reduced the number of reticulations from eight to two in the species network (cp. Figures 3B with 3D). The two conflicting splits within the eta-theta-zeta-epsilon cluster are very likely due to the recombination within the nuclear rDNA cistron dataset (PhiTest, *p* = 0.0018). As expected, no recombination was detected within the mitochondrial COI dataset (PhiTest, *p* = 0.1261).

The presence of introgression during the evolutionary history of plagiotomids was tested also with the software JML ver. 1.3.1. This program uses posterior checking to test whether the minimum distance between sequences of two species is smaller than expected under a scenario that does not account for hybridization. Potential hybridization within the rDNA cistron dataset was detected between lineages beta and eta (*p* = 0.0150), beta and theta (*p* = 0.0224), beta and zeta (*p* = 0.0416), beta and epsilon (*p* = 0.0157), delta and theta (*p* = 0.0048), as well as between gamma and theta (*p* = 0.0048). As inferred from the COI dataset, lineage alpha was involved in potential hybridization with all other lineages of the *P. lumbrici* complex: alpha and beta (*p* = 0.0002), alpha and gamma (*p* = 0.0105), alpha and delta (*p* = 0.0002), alpha and eta (*p* = 0.0002), alpha and theta (*p* = 0.0187), alpha and zeta (*p* = 0.0263), alpha and epsilon (*p* = 0.0220).

To summarize, no traces of hybridization between *P. aporrectodeae* sp. n. and any lineage of the *P. lumbrici* complex were detected. On the other hand, all lineages of the *P. lumbrici* complex very likely experienced hybridization episodes during their evolutionary history. Despite that, only 16 out of the 115 specimens analyzed (13.91%) were identified to potentially have a hybrid origin.

### Variability in the rDNA cistron of plagiotomids

**Figure 5:**
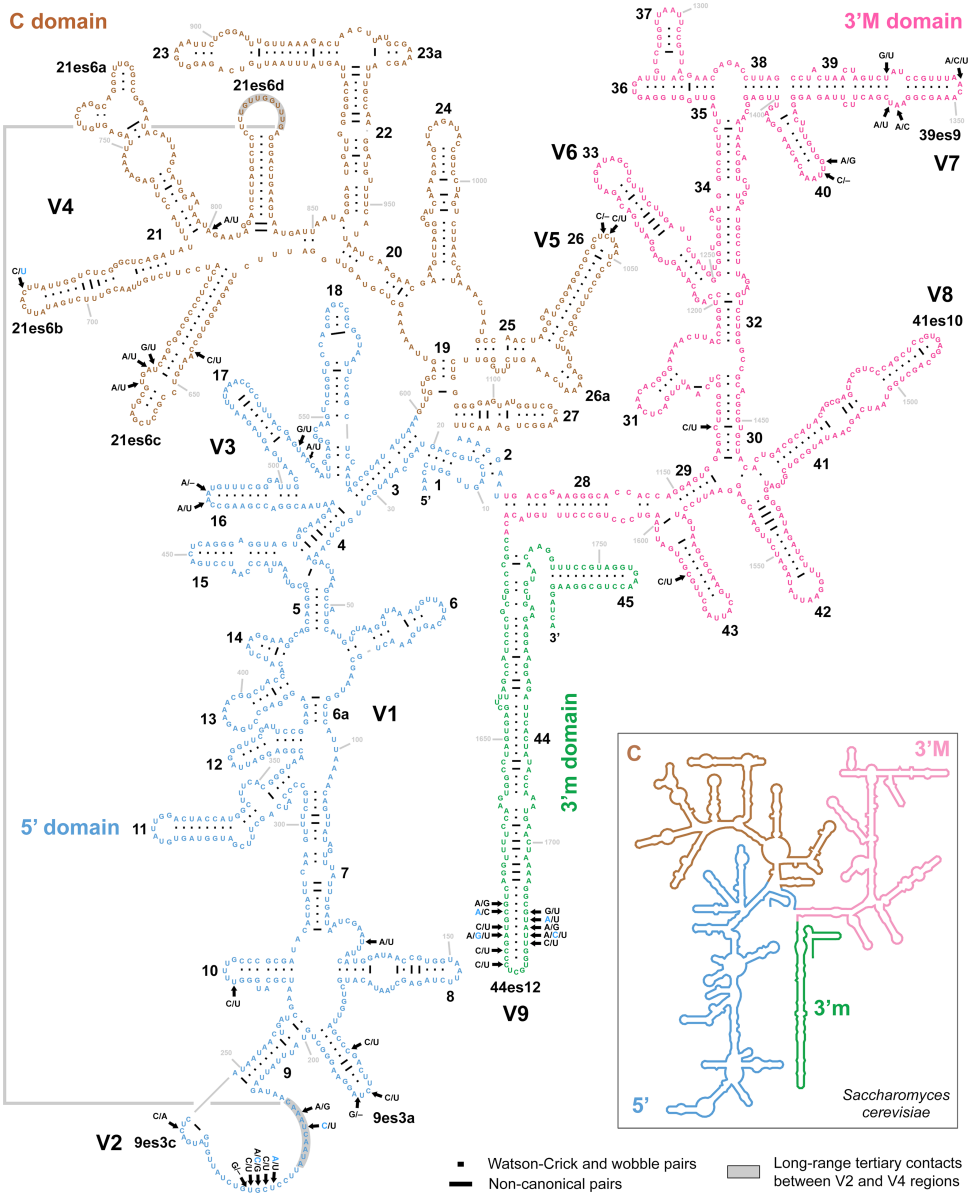
Secondary structure of the 18S rRNA molecule of specimen PUz 38 LT from the *P. lumbrici* complex. Variable nucleotide positions are marked by thick arrows and diagnostic molecular autapomorphies of *P. aporrectodeae* sp. n. are denoted by blue color. Variable nucleotide positions were localized in the V2–V5, V7, and V9 regions of the 18S rRNA molecule. Mutations tended to become accumulated in the single-stranded region of V2 and the double-stranded region of helix 44es12 of V9. There were as many as 42 polymorphic positions, whereas 13 were situated in terminal loops of helices, six in bulges or adjacent positions, nine in single-stranded regions, and 14 in double-stranded regions. Note the two compensatory base changes in the V2 region involved in long-range tertiary contacts with helix 21es6d in the V4 region. The 18S secondary structure map of *Saccharomyces cerevisiae* (inset) is from http://apollo.chemistry.gatech.edu/RibosomeGallery.

As mentioned above, no variability was detected in the rDNA cistron of *P. aporrectodeae* sp. n., while unusually high variability (up to 1.88%) was observed within the *P. lumbrici* complex. Variable nucleotide positions were localized in the V2–V5, V7, and V9 regions of the 18S rRNA molecule (Figure 5). More specifically, out of the 42 polymorphic positions, 13 were situated in terminal loops of helices, six in bulges or adjacent positions, nine in single-stranded regions, and 14 in double-stranded regions. Mutations in the double-stranded regions were typically involved in compensatory base changes (CBCs) or retained helical structure when involved in non-canonical pairings (helix 30). Interestingly, two CBCs were found also in the V2 region mediating a long-range tertiary contact with helix 21es6d from the V4 region (Figure 5). Mutations tended to become accumulated in the single-stranded region of V2 and the double-stranded region of helix 44es12 of V9.

**Figure 6:**
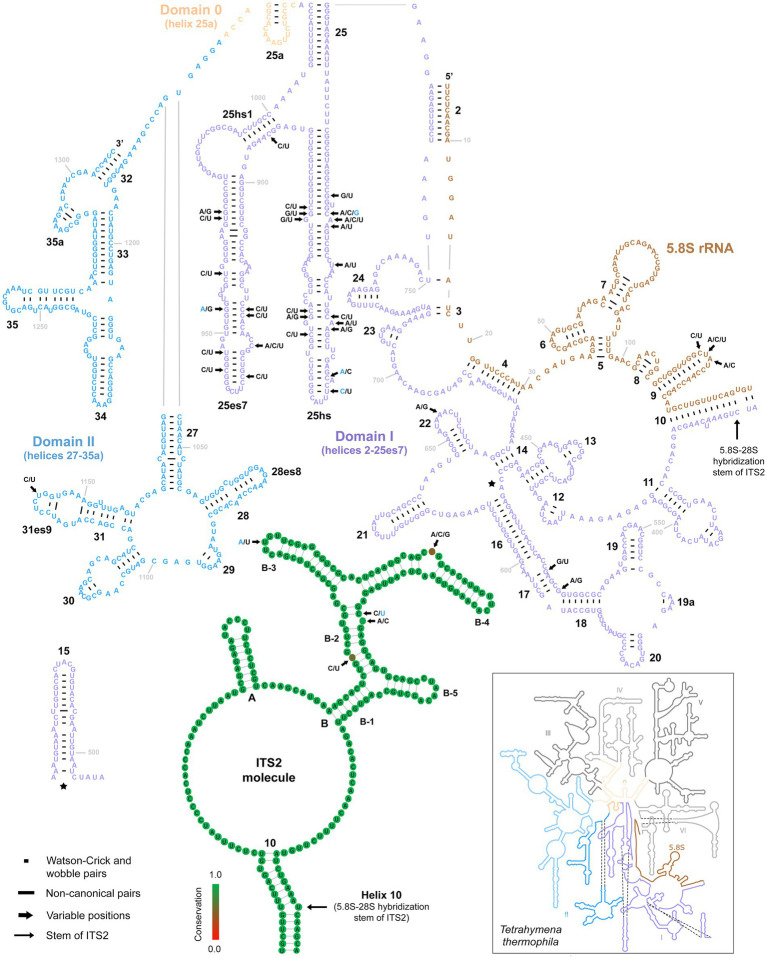
Secondary structure of the 5.8S rRNA molecule and the first two domains of the 28S rRNA molecule of specimen PUz 38 LT from the *P. lumbrici* complex as well as the consensus secondary structure of the ITS2 molecules of 115 *Plagiotoma* specimens. Variable nucleotide positions are marked by thick arrows and diagnostic molecular autapomorphies of *P. aporrectodeae* sp. n. are denoted by blue color. Only three variable positions were present in the 5.8S rRNA molecule and all were accumulated in the terminal loop of helix 9. Internal transcribed spacer 2 was similarly conservative, harboring only five polymorphic positions. Finally, there were as many as 31 mutations in the first two barcoding domains of the 28S rRNA molecule. All but one were situated in the first domain. The 5.8S-28S secondary structure map of *Tetrahymena thermophila* (inset) is from http://apollo.chemistry.gatech.edu/RibosomeGallery.

Only three variable positions were present in the 5.8S rRNA molecule (Figure 6, upper right panel). All were accumulated in the terminal loop of helix 9. Internal transcribed spacer 2 was similarly conservative, harboring only five polymorphic positions: one in the terminal loop of sub-helix B-3, one in the bulge of sub-helix B-4, and three in sub-helix B-2 (Figure 6, lower middle panel). There were as many as 31 mutations in the first two barcoding domains of the 28S rRNA molecule. All but one were situated in the first domain, whereby as many as 27 polymorphic sites were detected in the hypervariable helix 25. Interestingly, about 65% of these mutations were involved in CBCs (Figure 6).

### Morphological variability and delimitation of groups in plagiotomids

**Figure 7:**
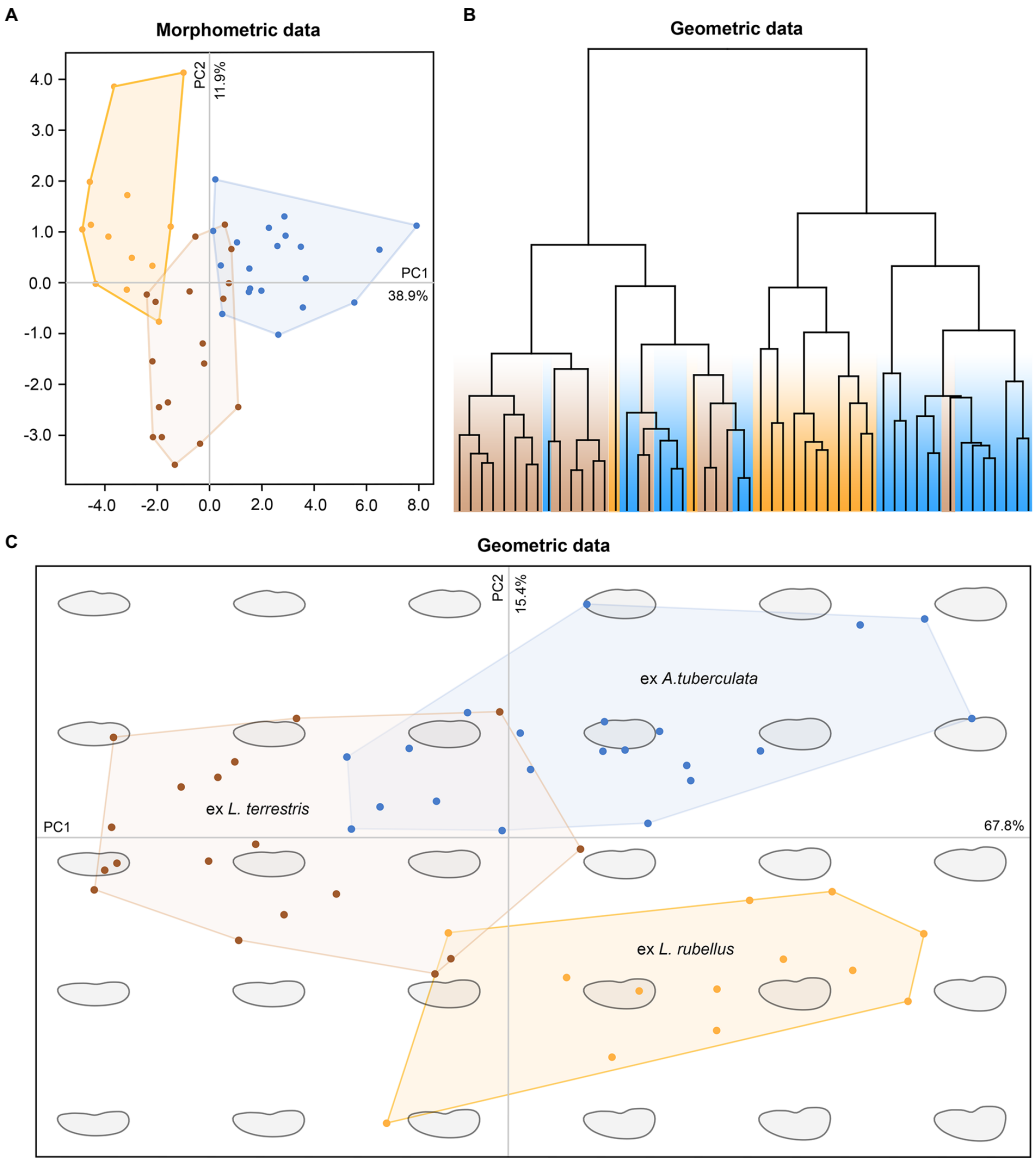
Multivariate statistical analyses of morphometric and geometric data of 52 protargol-impregnated specimens originated from epigeic (marked by yellow color), anecic (brown), and endogeic (blue) lumbricid earthworms. **(A)** Ordination diagram based on principal component analysis of 21 morphometric characters. **(B)** Cladogram based on geometric information and constructed using the complete linkage algorithm. **(C)** Ordination diagram based on principal component analysis of the Fourier coefficients derived from geometric information.

The pronounced genetic variability provoked a question of whether plagiotomids isolated from the three ecological groups of lumbricid earthworms could be also delimited morphologically. However, any qualitative morphological feature that would enable their discrimination was not recognized. To assess the utility of morphometric features in delimitation of the three ecological groups of plagiotomids, a multidimensional statistical approach including principal component analysis (PCA) and elliptical Fourier analysis of body shape was utilized. Although some trends were recognizable in the PCA ordination diagram based on 21 morphometric characters, only plagiotomids living in the epigeic *L. rubellus* could be unambiguously separated from those occurring in the endogeic *A. tuberculata* (Figure 7A). The following morphometric features most contributed to the distinction of these two clusters along the first ordination axis: body length (loading 0.3295), body width (0.3047), and nuclear figure length (0.3168). Plagiotomids isolated from the anecic *L. terrestris* were partially mixed with those from *L. rubellus* and *A. tuberculata*. Nevertheless, *L. terrestris*-dwelling plagiotomids displayed a trend to separate from the other two groups along the second ordination axis, which reached the highest correlations with the length/width ratio of the anteriormost (0.3827) and posteriormost (0.4962) macronuclear nodule as well as with the ratio of the adoral zone to the body length (−0.3498). In other words, plagiotomids inhabiting the digestive tube of epigeic earthworms tend to have smaller and narrower bodies as well as a shorter nuclear figure and adoral zone of membranelles. On the other hand, plagiotomids colonizing endogeic earthworms are larger on average, possessing a higher number of adoral organelles and a longer nuclear figure. Plagiotomids living in anecic earthworms are morphometrically intermediate.

Shape analyses brought very similar results as did morphometric analyses. More specifically, plagiotomids isolated from the three ecological groups of earthworms were intermingled in the hierarchical cluster analysis based on Euclid distance and complete linkage algorithm (Figure 7B), and plagiotomids isolated from the epigeic *L. rubellus* could be discerned from those isolated from the endogeic *A. tuberculata* in the PCA diagram based on the Fourier coefficients (Figure 7C). Statistically significant differences among shapes of the three ecological groups of plagiotomids were also corroborated by MANOVA performed on the PCA objects (Hotelling-Lawley trace = 48.25, approximate *F*_55, 382_ = 67.03, *p* = 2.2e–16). Thus, plagiotomids isolated from epigeic earthworms tend to be narrower and more reniform, while those from endogeic earthworms are more stocky and oblong. The cell outline of plagiotomids living in anecic earthworms resembles that of plagiotomids inhabiting epigeic earthworms but is less reniform.

To summarize, the plagiotomid lineages as delimited by molecular data cannot be unambiguously recognized either by qualitative, morphometric (Figure 7A), or cell geometric data (Figures 7B,C). There is a continuum of variability among plagiotomids isolated from the three ecological groups of earthworms. Plagiotomids thus form a single highly variable cluster in the phenotypic space.

## Taxonomic account

The present phylogenetic analyses suggested the existence of nine mitochondrial plagiotomid lineages in the digestive tract of the three lumbricid earthworm species investigated. However, only one of these lineages occupies an adaptive zone (i.e., the digestive tube of *Aporrectodea*) different from that of other lineages (i.e., the digestive tube of *Lumbricus*) and evolves separately from them. We endow this lineage with a new name, *P. aporrectodeae* sp. n. The remaining eight lineages are assigned to the *P. lumbrici* species complex. Because traces of hybridization and/or recombination have been detected within this complex, we found the speciation processes of these eight lineages to be incomplete and hence we prefer to not name them.

Because *P. aporrectodeae* sp. n. cannot be unambiguously separated from the *P. lumbrici* complex with morphological data (Figures 7A–C), we use also molecular information to diagnose the new species. We interpret the isolated DNA as type material of the new species, which conforms to Article 72.5.1 of the International Commission on Zoological Nomenclature (1999). Following Recommendation 8A of the International Commission on Zoological Nomenclature (2012), this work was registered in ZooBank (urn:lsid:zoobank.org:pub:5C08FE03-FEBF-4537-9413-841CC53316A2).

### *Plagiotoma aporrectodeae* Sp. N. (Figures 2A,B, Figures 4A,B, Figures 8A–I, Figures 9A–H)

#### Zoobank registration number of new species

urn:lsid:zoobank.org:act:E180480A-EE44-47E3-9198-295AC983D4E6.

**Figure 8:**
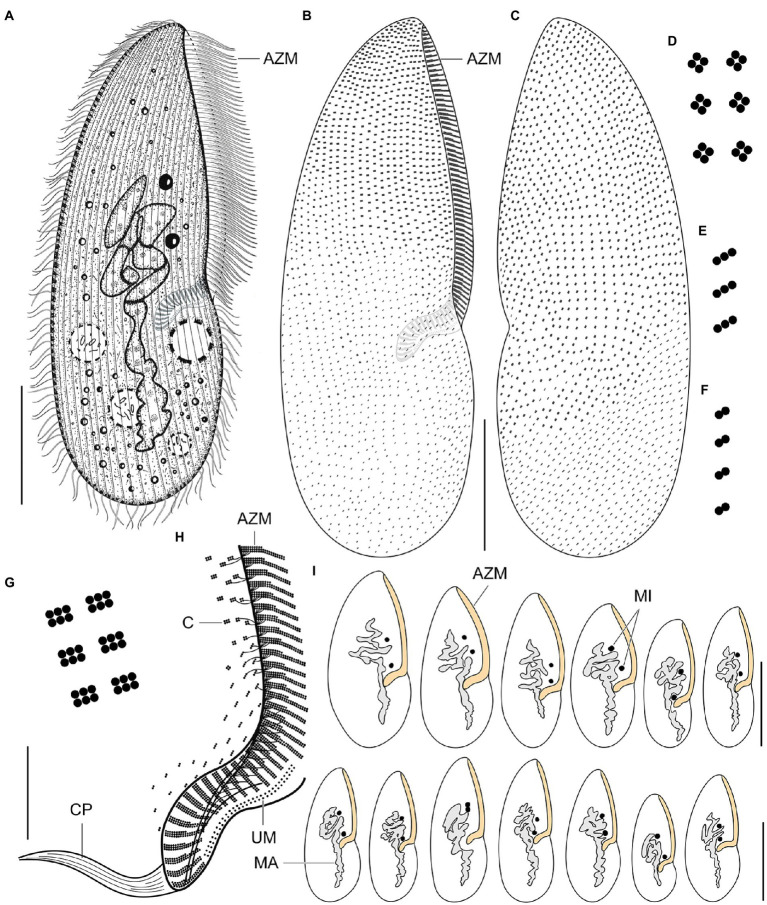
*Plagiotoma aporrectodeae* sp. n., type population from life **(A)** and after protargol impregnation **(B–I)**. **(A)** Ventral view of a representative specimen. The body is almost elliptical to narrowly elliptical, with a more or less pointed anterior end and a broadly rounded posterior end. The nuclear apparatus consists of 14–23 macronuclear nodules and invariably two micronuclei. The single contractile vacuole is localized posterior to the buccal vertex. **(B, C)** Cirral pattern of ventral and dorsal sides. Somatic ciliature is “holotrichous”-like, as cirri are indistinct and unspecialized. There are 18–25 ventral and 20–26 dorsal longitudinal cirral rows. **(D–G)** Fine structure of cirri in the anterior body region **(G)**, mid-body **(D)**, and posterior body region **(E, F)**. **(H)** Detail showing the oral ciliature. Adoral zone occupies about 51–67% of body length and consists of 65–77 membranelles, forming a *Gonostomum*-like pattern. **(I)** Variability of body shape and size as well as of the nuclear (shaded gray) and oral (shaded yellow) apparatus. AZM, adoral zone of membranelles; C, cirrus; CP, cytopharynx; MA, macronucleus; MI, micronuclei; UM, undulating membranes. Scale bars = 10 μm **(H)**, 50 μm **(A–C)**, and 100 μm (I).

**Figure 9:**
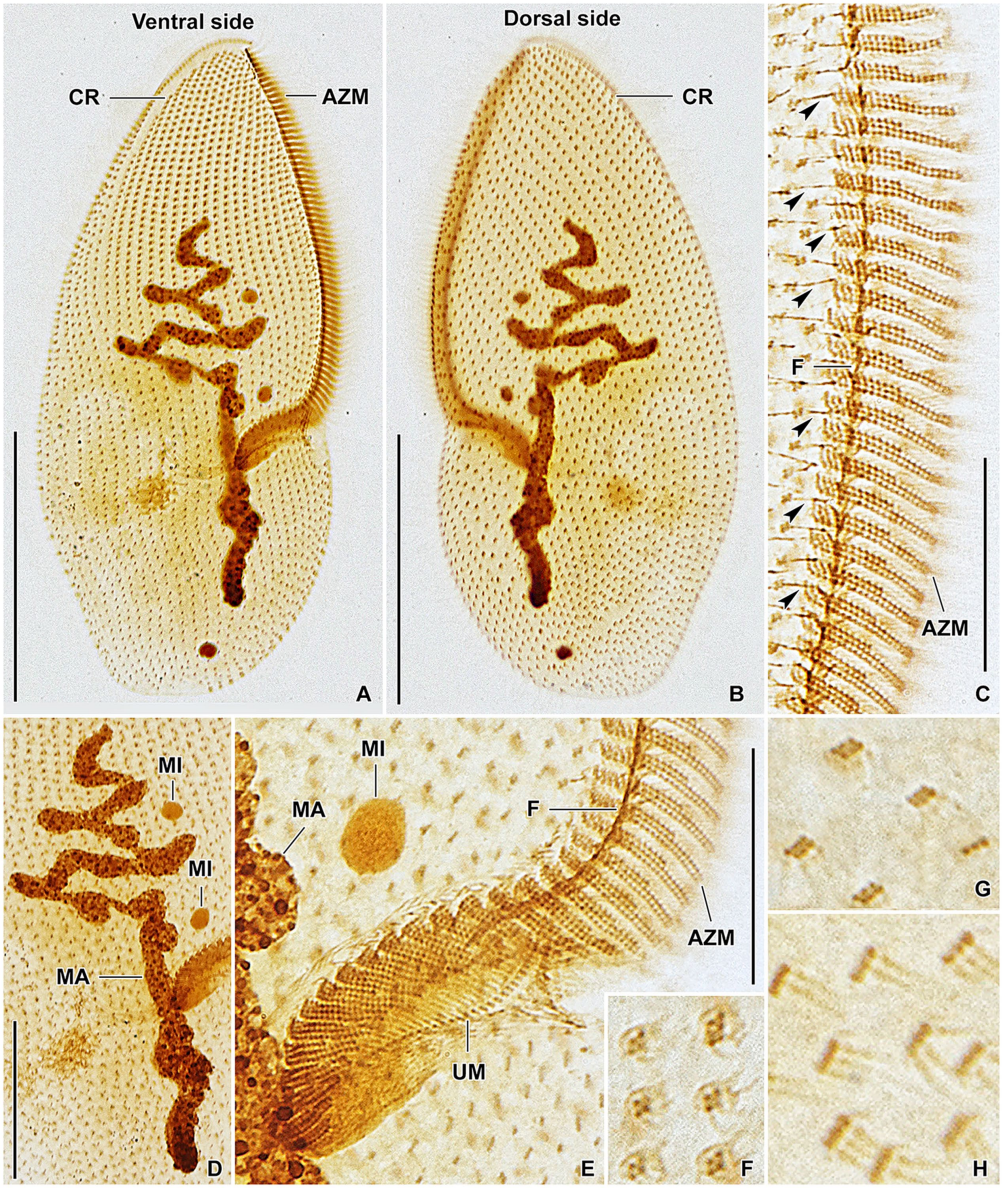
*Plagiotoma aporrectodeae* sp. n., type population after protargol impregnation. **(A, B)** Cirral pattern of ventral and dorsal sides. Somatic ciliature is “holotrichous”-like, as cirri are indistinct and unspecialized. There are 18–25 ventral and 20–26 dorsal longitudinal cirral rows. **(C)** Detail showing the fine structure of the adoral zone of membranelles. Individual adoral membranelles are composed of three horizontal rows basal bodies. The anterior row is distinctly shorter than the two other rows. Arrowheads denote fibres connecting cirri with the submembranellar fibre bundle. **(D)** The nuclear apparatus consists of a branched macronuclear strand and two globular micronuclei. **(E)** Detail showing the fine structure of the proximal region of the adoral zone of membranelles. Adoral zone occupies about 51–67% of body length and consists of 65–77 membranelles, forming a *Gonostomum*-like pattern. **(F–H)** Fine structure of cirri in the anterior body region **(G)**, mid-body **(F)**, and posterior body region **(H)**. AZM, adoral zone of membranelles; CR, cirral rows; F, submembranellar fibre bundle; MA, macronucleus; MI, micronuclei; UM, undulating membranes. Scale bars: 20 μm **(C, E)**, 50 μm **(D)**, and 100 μm **(A, B)**.

#### Morphological diagnosis

Body size about 165–245 × 60–110 μm, with an average of 190 × 75 μm. Shape almost elliptical to narrowly elliptical with a length:width ratio 2.1–3.3:1, anterior end almost pointed, while posterior end broadly rounded. Nuclear apparatus usually about 55 μm apart from anterior body end, approximately 110 μm long after protargol impregnation, terminating distinctly posterior to proximal end of adoral zone of membranelles; consists of 14–23 macronuclear nodules and invariably two micronuclei. Single contractile vacuole near cytostome. Cortex rigid, cortical granules absent. Somatic ciliature “holotrichous”-like, as cirri indistinct and unspecialized; 18–25 ventral and 20–26 dorsal longitudinal cirral rows. Adoral zone occupies about 51–67% of body length and consists of 65–77 membranelles, forming a *Gonostomum*-like pattern.

#### Molecular diagnosis

The following combination of nucleotide characters unambiguously defines the new species. rDNA cistron (ordinal numbers of positions are followed by particular nucleotide autapomorphies): 219 C, 229 A, 231 C, 714 T, 1678 A, 1681 G, 1693 C, 1695 A, 1809 C, 1810 T, 2127 A, 2187 T, 2691 G, 2720 A, 2723 C, 2841 A. All diagnostic molecular autapomorphies are marked by arrows and blue color in Figures 5, 6. COI (codon ordinal numbers are followed by the corresponding span of nucleotide positions in parentheses): 15 (43–45) GCT, 49 (145–147) ACA, 76 (226–228) GCA, 100 (298–300) TAC, 124 (370–372) ATC, 125 (373–375) ATA, 130 (388–390) GGT, 137 (409–411) TCA, 147 (439–441) CGA.

#### Type locality

A meadow near the River Danube, Devín, Bratislava, Slovak Republic, 48°10′15.3″N 16°59′04.3″E.

#### Type host

*Aporrectodea tuberculata* (Eisen, 1874).

#### Type material

A DNA sample of the holotype specimen (LS 43 AT) has been deposited in Natural History Museum, Vajanského nábrežie 2, 810 06 Bratislava, Slovakia (ID Collection Code 01426296).

#### Gene sequences

The 18S rRNA gene, ITS region-28S rRNA gene, and COI sequences of the holotype specimen have been deposited in GenBank under the following accession nos. OP538924, OP539039, and OP562346, respectively.

#### Etymology

The specific epithet is a singular genitive case of the Neo-Latin noun *Aporrectode*·*a*, ·*ae* [f], meaning a *Plagiotoma* from *Aporrectodea*. The species-group name is to be treated as an adjective used as a substantive in the genitive case, because of its derivation from the host’s generic name (Article 11.9.1.4. of the International Commission on Zoological Nomenclature, 1999).

#### Taxonomic status and comparison with congeners

The new species has a very similar overall morphology as members of the *P. lumbrici* complex. They match, *inter alia*, in body shape and size, the nuclear and contractile vacuole apparatus, and the cirral pattern (cp. Figures 8A–I, 9A–H with Supplementary Figures S3A–H, 4A–H). Reliable identification and separation from the *P. lumbrici* complex thus require molecular data and information about the earthworm host species. It might be, therefore, assumed that *P. aporrectodeae* should be rather classified as a subspecies of *P. lumbrici*. According to Zusi (1982), subspecies serve to “collect” the infraspecific variation. This is apparently not the case of *P. aporrectodeae*, which can be genetically and ecologically unambiguously separated from the *P. lumbrici* complex. No traces of hybridization and/or recombination of *P. aporrectodeae* with any member of the *P. lumbrici* complex were detected, documenting that there is no gene flow between these two biological entities. According to Mayr’s (1995) biological species concept, *P. aporrectodeae* can be considered a distinct species, since it is reproductively isolated from the *P. lumbrici* complex. The distinctness of *P. aporrectodeae* from the *P. lumbrici* complex is further strengthened by as much as 1.06–2.00% in the nuclear rDNA cistron and 8.77–11.70% in the COI gene. Such profound differences both in the highly conservative nuclear and fast-evolving mitochondrial genes can hardly be considered as an infraspecific variation. There are, indeed, multiple symbiotic ciliate species that cannot be unambiguously separated morphologically but are well delimited using multi-gene and ecological data (Lynn et al., 2014, 2018; Obert and Vďačný, 2019; Pecina and Vďačný, 2020, 2022; Obert et al., 2021; Rataj and Vďačný, 2021). In this light, we prefer to classify *P. aporrectodeae* as a distinct species and not as a subspecies within the *P. lumbrici* complex.

Hitherto, only two further species have been described in the genus *Plagiotoma*: *P. africana* Albaret and Njiné, 1975 and *P. dichogasteri* Mandal and Nair, 1975. Unfortunately, no molecular data are available for these two species and the earthworm host of *P. africana* is not known. *Plagiotoma aporrectodeae* differs from *P. africana* by a much larger body (165–245 × 60–110 μm vs. 77–137 × 29–55 μm) as well as by a higher number of adoral membranelles (65–77 vs. 60) and micronuclei (2 vs. 1; Albaret, 1975; Albaret and Njiné, 1975). Our new species could be distinguished from *P. dichogasteri* by the body size (165–245 × 60–110 μm vs. 63–105 × 23–38 μm), the number of cirral rows (18–25 ventral and 20–26 dorsal rows vs. 11–13 rows on each body side), the number of macronuclear nodules (14–23 vs. 8–13), and by the host (the lumbricid *Aporrectodea tuberculata* vs. the octochaetid *Dichogaster bolaui* (Michaelsen, 1891)) (Mandal and Nair, 1975).

## Discussion

### Diversity of plagiotomids in lumbricid earthworms

For over 180 years, all plagiotomids isolated from lumbricid earthworms were consistently identified as a single species, *P. lumbrici*. This obligate gut endosymbiont was reported from three biogeographic realms: the Palearctic (Dujardin, 1841; Pertzewa, 1929; Heidenreich, 1935; de Puytorac and Mauret, 1956; Dworakowska, 1966; Albaret, 1973, 1975), the Nearctic (Affaa et al., 2004), and the Neotropic (Cordero, 1928). Literature data thus suggests a wide distribution for *P. lumbrici* on one hand and low species differentiation within the genus *Plagiotoma* on the other hand. In accordance with this traditional view, the present multivariate statistical analyses of morphometric and cell geometric data did not reveal any well-delimited and discrete unites in the phenotypic space but a continuum of variability among plagiotomids isolated from the three ecological groups of lumbricid earthworms (Figures 7A–C). These findings, however, contrast with the high molecular diversity detected within the morphospecies *P. lumbrici* (Figures 5, 6) already at mesoscale, whereby as many as nine distinct mitochondrial lineages were identified in SW Slovakia (Figures 2B, 3C,D, 4A,B). We remain conservative and recognize, using Mayr’s (1995) biological species concept and Van Valen’s (1976) ecological species concept, only two discrete biological entities: the *P. lumbrici* complex comprising eight mitochondrial lineages and *P. aporrectodeae* sp. n. with just a single mitochondrial lineage. This, however, does not mean that the plagiotomid diversity is low. On the contrary, it is comparatively high but obscured by a complex evolutionary history shaped by secondary contacts of lineages whose speciation processes were not completed (see below).

Despite the hidden diversity, there are still pronounced differences (of at least one or two orders of magnitude) in alpha-diversities of plagiotomids and other endosymbiotic ciliate groups (nyctotherids, astomes, and hysterocinetids) living in the digestive tube of earthworms. A plausible explanation for this diversity paradox might be the differing mating-type system. Plagiotomids belong to the class Spirotrichea, which has a ‘high-multiple (open)’ type. This mating system strongly favors cross-mating phenomena, causing the genetic boundaries between species living in sympatry to be relaxed (e.g., Nobili et al., 1978; Valbonesi et al., 1988; Kuhlmann and Sato, 1993; Di Guiseppe et al., 2022). On the other hand, astomes and hysterocinetids belong to the class Oligohymenophorea whose mating system is ‘low-multiple (closed)’ (Orias et al., 2017). The closed system permits the establishment of strong species boundaries by strengthening incompatibilities among populations of different mating types. Mating incompatibilities fuel speciation processes by limiting gene flow, which supports evolutionary cohesion and ultimately leads to a comparatively low genetic variability within species. This hypothesis is corroborated by our observations on astomes (Obert and Vďačný, 2019, 2020a, 2021; Obert et al., 2021) and hysterocinetids (manuscript in preparation). By contrast to plagiotomids, individual species of astomes and hysterocinetids are homogenous in the nuclear as well as mitochondrial rDNA cistron and exhibit up to 1% variability in the barcoding COI gene. The ‘closed’ mating system thus might be responsible for the comparatively high alpha-diversity of astomes and hysterocinetids (*cf.* de Puytorac, 1954, 1969, 1972; Raabe, 1972; Obert et al., 2021), while the ‘open’ system might be accountable for the creation of highly variable complexes in plagiotomids.

### Coevolution of plagiotomids with lumbricid earthworms

The evolution of lumbricid earthworms has been largely driven by paleoclimatic and paleogeographic events (e.g., Pérez-Losada et al., 2011; Fernández et al., 2013; Domínguez et al., 2015; James et al., 2021). Central Europe is the ancestral area for a large portion of known lumbricid genera, whose diversification was very likely shaped by range expansion and retraction due to global climatic changes from the Paleocene–Eocene epochs to the Pleistocene (Marchán et al., 2021). Both epigeic and anecic earthworms evolved multiple times from endogeic ancestors, which suggests subsoil-dwelling, temporary and horizontally oriented burrow systems, and geophagy (feeding on soil) to represent the ground pattern of lumbricid earthworms (Domínguez et al., 2015). Interestingly, not only plagiotomids but also astomes inhabiting endogeic earthworms form deep-branching clades (Obert and Vďačný, 2019, 2020a, 2021; Obert et al., 2021). This suggests that endogeic earthworms might have served as ancestral hosts for intestinal ciliates and both anecic and epigeic earthworms were colonized later.

Plagiotomid mitochondrial lineages exhibit a distinct host-correlated pattern (Figures 4A,B,E), which also resembles the situation in astomes associated with lumbricid earthworms (Obert and Vďačný, 2019, 2020a, 2021; Obert et al., 2021). Diversification of plagiotomids was thus very likely governed by adaptive radiation in connection with the ecological group of their earthworm hosts. A similar diversification mode was suggested also for astome ciliates inhabiting lumbricid earthworms (Obert et al., 2021). The matter is, however, much more complex in plagiotomids than in astomes. The present phylogenetic trees indicate at least two independent transfers of plagiotomids from the anecic to the epigeic earthworms as well as multiple duplication events without host switching (Figures 4A,B). The latter coevolutionary event was very likely enabled by alternating glacial–interglacial periods, which led to the separation and reconnection of *Lumbricus* populations in the Palearctic. The approximately 41,000–100,000-year climatic cycles might have allowed the formation of distinct mitochondrial plagiotomid lineages in spatially isolated populations of earthworms but were not long enough to enable the evolution of strong species boundaries. After the secondary contact of earthworms during interglacial periods, hybridization of their plagiotomid endosymbionts might have taken place, as suggested by the present split decomposition (Figure 3A), species network (Figure 3B), and JML analyses. Introgression might have ultimately dissolved the emerging species boundaries and led to the collapse of multiple plagiotomid lineages into a single highly variable biological entity (*cf.* Bog et al., 2017; Kearns et al., 2018).

According to the time-calibrated phylogenies, *Lumbricus* and *Aporrectodea* s.s. are sister taxa that diverged about 65 Ma, whereby each genus further radiated about 20–40 Ma (Domínguez et al., 2015). According to the present phylogenetic and JML analyses, there are no signs of introgression between *P. aporrectodeae* sp. n. isolated from *Aporrectodea* and the *P. lumbrici* complex isolated from *Lumbricus*. Their separation is thus completed and very likely dates to the *Aporrectodea–Lumbricus* split in the Paleocene–Eocene. *Plagiotoma aporrectodeae* sp. n. differs from members of the *P. lumbrici* complex by 1.06–2.00% in the rDNA cistron, which corresponds to 1.58–2.99 × 10^−4^ substitutions per site per one million years. This clock rate matches quite well estimates for the 18S rRNA gene in various groups of ciliates, ranging from 1.24–3.96 × 10^−4^ substitutions per site per one million years (Wright and Lynn, 1997; Vďačný, 2015; Vďačný et al., 2019). On the other hand, the maximum distance in the rDNA cistron within the *P. lumbrici* complex is up to 1.03%, which corresponds to a divergence time of about 35 Ma. This estimate falls almost perfectly in the period of the *Lumbricus* radiation (Domínguez et al., 2015). Separation of the eight mitochondrial lineages within the *P. lumbrici* complex was, however, not completed during the Oligocene–Pleistocene epochs very likely due to the episodic reconnection of various *Lumbricus* populations/species during interglacial periods. Secondary contacts of earthworms might have enabled episodic hybridization of their plagiotomid symbionts, which in turn led to the weakening of the emerging species boundaries. Thus, the range dynamics of the earthworm hosts very likely strongly affected the diversification processes of their ciliate endosymbionts.

### Effect of hybridization on diversity of plagiotomids

Hybridization is an important evolutionary process that affects diversification in various ways. It can accelerate diversification *via* formation of hybrid species (e.g., Stankowski and Streisfeld, 2015; Sefc et al., 2017; Ebersbach et al., 2020; White et al., 2020), but it can also slow down diversification *via* breakdown of species reproductive barriers, followed by fusion of previously separated lineages (e.g., Huxel, 1999; Gómez et al., 2015; Todesco et al., 2016). Organisms that were impacted by historical climate changes provide an excellent model system for studying how gene flow can drive but also prevent speciation. The evolution and distribution of lumbricid earthworms in the Palearctic were shaped by the Paleocene–Eocene Thermal Maximum and the Mid-Pleistocene Revolution, a fundamental change in the behavior of glacial cycles during the Quaternary glaciations (*cf.* Marchán et al., 2021). Repeated environmental oscillations thus might have enabled previously separated earthworm populations to come into secondary contact and hence allowed hybridization of their plagiotomid endosymbionts.

The present phylogenetic analyses revealed that eight out of the nine plagiotomid lineages show signs of hybridization (Figures 2A,B). All potential hybrids were detected only in two *Lumbricus* species, nevertheless, hybrids represented only 16 out of the 115 specimens analyzed (13.91%). The plagiotomid mitochondrial lineages differ by as much as 4.27–10.29% in the barcoding COI gene. Intra-species divergences in most ciliate species, however, typically range between 0 and 1% and only rarely are slightly higher than 1% (e.g., Kher et al., 2011, Doerder, 2014, 2019; Rataj and Vďačný, 2019, 2020, 2022; Obert et al., 2021; Zhang and Vďačný, 2021a,b, 2022). Doerder (2014) proposed a 4% interspecific divergence threshold for COI and Kher et al. (2011) even a more conservative 5% threshold. COI has been, indeed, recognized as a reliable barcoding marker in a variety of ciliates (e.g., Chantangsi et al., 2007; Strüder-Kypke and Lynn, 2010; Zhao et al., 2016; Park et al., 2019). In this light, all plagiotomid mitochondrial lineages could be classified as distinct molecular species. Despite this, we have found traces of hybridization and/or recombination among all lineages of the *P. lumbrici* complex (Figures 2A,B). These findings document that their speciation processes were not completed and introgression dissolved their emerging species boundaries. We, therefore, argue that gene exchange might have slowed down and ultimately hampered the diversification of *Lumbricus*-dwelling plagiotomids. In other words, hybridization led to their collapse into a single biological entity.

Hybridization is very likely responsible also for weakening the host structural specificity of plagiotomids by increasing their ecological amplitudes. This is documented by lineage delta, which was found both in *L. terrestris* and *L. rubellus* (Table 1). According to Albaret’s (1975) review, *P. lumbrici* has been so far isolated from four genera of the earthworm family Lumbricidae Rafinesque-Schmaltz, 1815: *Lumbricus* Linneus, 1758; *Nicodrilus* Bouché, 1972; *Aporrectodea* Orley, 1885; and *Scherotheca* Bouché, 1972. Specifically, it was recorded in four species of the genus *Lumbricus* [*L. castaneus* (Savigny, 1826); *L. friendi* Cognetti, 1904; *L. rubellus* Hoffmeister, 1843; and *L. terrestris* Linneus, 1758]; in two species of *Nicodrilus* [*N. giardi* (Ribaucourt, 1901) and *N. longus* (Ude, 1885), which is now considered a synonym of *Aporrectodea longa* (Ude, 1885)]; and in *S. savignyi* (Guerne and Horst, 1893). Although the molecular identity of plagiotomids reported in the literature is not known, all aforementioned reports are only from anecic and epigeic earthworms and hence might, indeed, refer to the *P. lumbrici* complex. This would suggest a rather broad host range and comparatively weak structural host specificity.

A further possible by-product of hybridization events might be the pronounced diversity (up to 1.88%) in the nuclear rDNA cistron within the *P. lumbrici* complex (Figures 5, 6). On the other hand, no signs of hybridization were noted in *P. aporrectodeae* sp. n. and no variability was detected in its rDNA cistron. Similarly, we did not observe any potential hybrids and any or very low variability in the highly conservative nuclear rDNA cistron or the hyper-variable COI gene in astomes and hysterocinetids (Obert et al., 2021). These observations indicate that introgression might increase molecular variability by overcoming concerted evolution, a process that converts copies of a gene in a multigene family into the same copy.

## Conclusion

We provide here the first molecular evidence of introgression in wild populations of ciliates. The present multifaceted analyses suggested that hybridization might decrease the alpha-diversity by dissolving species boundaries, weaken the structural host specificity by broadening ecological amplitudes, and increase the nuclear rDNA variability by overcoming concerted evolution within the *P. lumbrici* species complex. In addition, our study provoked several exciting questions: (1) Is the presence of hybridization positively correlated with the ‘high-multiple (open)’ mating system; (2) Do the aforementioned effects of hybridization hold also for other symbiotic and free-living ciliate groups; and (3) How widespread is hybrization in ciliates?

## Data availability statement

The data presented in the study are deposited in the GenBank database (https://www.ncbi.nlm.nih.gov/nucleotide/), accession numbers OP538845‒OP538959, OP538960‒OP539074, and OP562267‒OP562381. Results of all analyses are included in this published article and Supplementary material. GenBank accession numbers of sequences used in phylogenetic analyses can be found in the Supplementary material.

## Author contributions

PV conceived and designed the study, drafted the manuscript, and undertook the interpretation of the results. TO and PV conducted the sampling. TO and TZ conducted the laboratory work. PV, IR, and TO analyzed data. All authors contributed to the article and approved the submitted version.

## Funding

This work was supported by the Slovak Research and Development Agency (grant number APVV-19-0076), the Grant Agency of the Ministry of Education, Science, Research and Sport of the Slovak Republic and Slovak Academy of Sciences (grant number VEGA 1/0013/21), and by Comenius University (grant number UK/228/2022).

## Conflict of interest

The authors declare that the research was conducted in the absence of any commercial or financial relationships that could be construed as a potential conflict of interest.

## Publisher’s note

All claims expressed in this article are solely those of the authors and do not necessarily represent those of their affiliated organizations, or those of the publisher, the editors and the reviewers. Any product that may be evaluated in this article, or claim that may be made by its manufacturer, is not guaranteed or endorsed by the publisher.

## Footnotes

^1^ https://inkscape.org/en/

^2^ http://CRAN.R-project.org/package=Momocs

^3^ http://tree.bio.ed.ac.uk/software/figtree/
