# Supplementary material for: First molecular evidence of hybridization in endosymbiotic ciliates (Protista, Ciliophora)
Source: Front Microbiol. 2022 Dec 8;13:1067315. doi: 10.3389/fmicb.2022.1067315 (PMC9772525; doi:10.3389/fmicb.2022.1067315)
Supplement: Supplementary file 1 [file Data_Sheet_1.PDF]

SUPPLEMENTARY MATERIAL

First molecular evidence of hybridization in endosymbiotic ciliates (Protista, Ciliophora)

Tomáš Obert, Tengyue Zhang, Ivan Rurik, Peter Vďačný*

Department of Zoology, Faculty of Natural Sciences, Comenius University in Bratislava, Bratislava, Slovakia

Correspondence:
Peter Vďačný
peter.vdacny@uniba.sk

**Pages:**	**17**
**Tables:**	**5**
**Figures:**	**4**
**References:**	**11**

Supplementary table S1. Characterization of collection sites of earthworm species examined for the presence of plagiotomids.

No.	Locality code	Collection date[a]	Collection site	GPS coordinates	Host species	No. of specimens
**Decomposing plant material**						
1	BZ	05/19/2018	Decomposing plant material from a compost heap, south part of the Botanical Garden, Karlova Ves, Bratislava	48°08′43.5″N 17°04′21.1″E	*Eisenia andrei*[b]	60
2	BZkv	09/12/2019	Decomposing plant material from a compost heap, west part of the Botanical Garden, Karlova Ves, Bratislava	48°08′51.0″N 17°04′26.5″E	*Eisenia andrei*	50
3	JA-1	06/28/2018	Decomposing plant material and humous soil from a garden compost heap, Jakubská ulica street, Rača, Bratislava	48°12′10.9″N 17°09′05.7″E	*Eisenia andrei*	60
4	NG	06/30/2018	Humous soil with high content of decomposing plant material from a garden at the foothill of the Malé Karpaty Mts., Na Grunte street, Nové mesto, Bratislava	48°11′44.4″N 17°07′47.5″E	*Dendrobaena veneta*	25
5	PUh	12/04/2019	Decomposing material under fallen trees, poplar forest in the vicinity of the Pusté Úľany village, Galanta district	48°13′08.2″N 17°34′43.0″E	*Lumbricus rubellus*	20
6	PUh	04/16/2022	Forest soil under fallen trees, poplar forest in the vicinity of the Pusté Úľany village, Galanta district	48°13′08.2″N 17°34′43.0″E	*Allolobophora chlorotica*	5
					Lumbricus rubellus	10
					Octolasion lacteum	5
					Octolasion sp.	5
**Floodplain and waterlogged soils**						
7	KR	10/03/2017	Floodplain soil from a riparian, willow-poplar forest near the Karlova Ves branch of the Danube river, Bratislava	48°08′47.5″N 17°04′08.0″E	*Lumbricus terrestris*	50
8	AMc	10/14/2019	Floodplain soil from a riparian, willow-poplar forest near the Danube river, Devín, Bratislava	48°10′46.0″N 16°58′39.6″E	*Aporrectodea tuberculata*	25
9	HkD	10/14/2019	Moist soil near the confluence of the Danube and Morava rivers, foot of the cliff of the Devín Castle, Bratislava	48°10′28.0″N 16°58′37.0″E	*Aporrectodea rosea*	5
					Aporrectodea tuberculata	15
10	LS	10/14/2019	Floodplain soil from a meadow near the Danube river, Devín, Bratislava	48°10′15.3″N 16°59′04.3″E	*Aporrectodea tuberculata*	20
					Octolasion lacteum	10
11	KDo	09/15/2019	Waterlogged soil around the Banský potok stream in an urban oak-hornbeam forest, Knižková dolina valley, Bratislava, Malé Karpaty Mts.	48°13′37.0″N 17°07′13.1″E	*Dendrobaena octaedra*	3
					Eiseniella tetraedra	10
12	KDo	09/29/2019	Moist soil in the vicinity of the Banský potok stream in an urban oak-hornbeam forest, Knižková dolina valley, Bratislava, Malé Karpaty Mts.	48°12′60.0″N 17°08′21.7″E	*Lumbricus terrestris*	1

13	CBk	10/06/2019	Waterlogged soil around the Šenkársky potok stream in an urban oak-hornbeam forest, Bratislava, Malé Karpaty Mts.	48°13′50.2″N 17°08′21.9″E	*Bimastos rubidus*	15
					Eiseniella tetraedra	20
					Octolasion sp.	30
14	MB	10/27/2019	Moist soil from an urban oak-hornbeam forest in the vicinity of Malá Baňa, Bratislava, Malé Karpaty Mts.	48°13′14.7″N 17°07′45.6″E	*Fitzingeria platyura*	1
15	PU	07/02/2018	Upper 50 cm turf layer in the riparian zone of the Rašelinisko pond in the vicinity of the Pusté Úľany village, Galanta district	48°13′21.9″N 17°34′49.9″E	*Octolasion tyrtaeum*	5
16	KL	04/29/2019	Waterlogged soil in the riparian zone of the River Danube, Kráľovská lúka	47°52′59.5″N 17°31′02.8″E	*Lumbriculus variegatus*	3
17	KpPP	05/02/2020	Waterlogged soil in the riparian zone of the Kráľov potok stream in the village of Plavecké podhradie, Malacky district	48°28′49.8″N 17°16′26.1″E	*Eiseniella tetraedra*	10
18	ST	09/24/2020	Hyporeal of the Stupavský potok stream, Stupava	48°16′38.2″N 17°02′25.0″E	*Criodrilus lacuum*	20
19	JJ	05/03/2021	Waterlogged soil around Jurské jazierko pond in an urban oak-hornbeam forest, district of the village of Svätý Jur, Malé Karpaty Mts.	48°15′28.0″N 17°09′14.6″E	*Eiseniella tetraedra*	5
					Lumbriculus variegatus	5
20	CVsk	04/06/2022	Waterlogged soil in the riparian zone of the Šúrsky kanál branch, Čierna voda river, Bratislava district	48°12′46.0″N 17°13′25.3″E	*Eiseniella tetraedra*	15
					Octodrilus cf. *gradinescui*	5
21	TR	04/06/2022	Waterlogged soil around and hyporeal of the euthrophic Trnávka stream, Trnava	48°22′01.1″N 17°35′26.2″E	*Aporrectodea trapezoides*	1
					Criodrilus lacuum	25
**Agricultural and grassland soils**						
22	RZ	06/06/2017	Agricultural soil from a garden, Šúrska ulica street, Rendez, Bratislava	48°11′57.6″N 17°10′25.0″E	*Lumbricus terrestris*	50
23	RZ	11/13/2021	Agricultural soil from a garden, Šúrska ulica street, Rendez, Bratislava	48°11′57.6″N 17°10′25.0″E	*Aporrectodea tuberculata*	10
24	JA-2	11/28/2018	Agricultural soil from a garden, Jakubská ulica street, Rača, Bratislava	48°12′12.2″N 17°09′03.1″E	*Allolobophora chlorotica*	10
25	FNS	12/09/2019	Soil from a grassland in the vicinity of the Faculty of Natural Sciences, Comenius University, Karlova Ves, Bratislava	48°08′56.7″N 17°04′21.2″E	*Lumbricus terrestris*	25
26	PUz	06/20/2018	Agricultural soil from a garden, Spodná ulica street, Pusté Úľany village, Galanta district	48°13′41.0″N 17°34′48.6″E	*Aporrectodea tuberculata*	20
27	PUz	09/14/2019	Agricultural soil from a garden, Spodná ulica street, Pusté Úľany village, Galanta district	48°13′41.0″N 17°34′48.6″E	*Allolobophora chlorotica*	10
					Aporrectodea trapezoides	10
					Aporrectodea tuberculata	20
					Lumbricus terrestris	20
28	PUp	11/28/2019	Agricultural soil from a field in the vicinity of the Pusté Úľany village, Galanta district	48°13′19.2″N 17°34′57.1″E	*Aporrectodea tuberculata*	20
29	TT	05/13/2021	Agricultural soil from a garden, Pálárikova ulica street, Trnava	48°22′18.0″N 17°35′55.9″E	*Lumbricus terrestris*	10
30	JV	02/19/2022	Grassland soil in the recreation area in the surroundings of the village of Vojka nad Dunajom	47°57′44.3″N 17°24′24.0″E	*Aporrectodea tuberculata*	5
					Lumbricus terrestris	5

**Garden soils**						
31	JA-2	06/28/2018	Loamy soil with fallen needles in the surroundings of a garden wall, Jakubská ulica street, Rača, Bratislava	48°12′12.2″N 17°09′03.1″E	*Lumbricus terrestris*	50
32	JA-3	09/18/2019	Soil from a garden, Jakubská ulica street, Rača, Bratislava	48°12′11.4″N 17°09′05.3″E	*Allolobophora chlorotica*	10
					Aporrectodea trapezoides	20
33	JA	11/13/2021	Soil from a garden, Jakubská ulica street, Rača, Bratislava	48°12′11.4″N 17°09′05.3″E	*Lumbricus terrestris*	1
34	HO	11/08/2019	Soil from a garden, Horská ulica street, Nové mesto, Bratislava	48°11′52.1″N 17°08′03.3″E	*Lumbricus terrestris*	15
35	MU	10/13/2019	Soil from a garden, Moskovská ulica street, Staré mesto, Bratislava	48°09′05.0″N 17°07′18.2″E	*Octolasion lacteovicinum*	20
36	BZ	05/19/2018	Soil from the eastern part of the Botanical Garden, Karlova Ves, Bratislava	48°08′41.9″N 17°04′24.6″E	*Lumbricus terrestris*	10

[a] Dates are given as mo/d/yr.

[b] Identified as *Eisenia fetida* by Obert and Vďačný (2019) based on morphological data. However, the identification within the *E. fetida* complex was specified to *E. andrei* given the mitochondrial COI and ND1 sequences by Obert et al. (2021).

Supplementary table S2. Primers used for amplification of molecular markers analyzed in plagiotomids and their earthworm hosts.

Molecular marker	Organism group	Primer name	Primer sequence (in 5' to 3' direction)	Reference
18S rRNA gene	Plagiotomids	Euk A	AAC CTG GTT GAT CCT GCC AGT	Medlin et al. (1988)
		Euk B	TGA TCC TTC TGC AGG TTC AC	Medlin et al. (1988)
ITS region and 28S rRNA gene[a]	Plagiotomids	ITS-F	GTA GGT GAA CCT GCG GAA GGA TCA TTA	Miao et al. (2008)
		LO-R	GCT ATC CTG AGR GAA ACT TCG	Pawlowski (2000)
Cytochrome *c* oxidase subunit I	Plagiotomids	CiCO1-Fv2	GWT GRG CKA TGA TYA CAC C	Park et al. (2019)
		CiCO1-Rv2	ACC ATR TAC ATA TGA TGW CC	Park et al. (2019)
	Earthworms	LCO 1490	GGT CAA CAA ATC ATA AAG ATA TTG G	Folmer et al. (1994)
		HCO 2198	TAA ACT TCA GGG TGA CCA AAA AAT CA	Folmer et al. (1994)
NADH-ubiquinone oxidoreductase chain 1	Earthworms	tRNA-Leu-ND1-LumbF2	GAA TAG TGC CAC AGG TTT AAA C	Pérez-Losada et al. (2009)
		tRNA-Leu-ND1-LumbR1b	TTA ACG TCA TCA GAG TTA TC	Pérez-Losada et al. (2009)

[a] The barcoding D1/D2 domains of the 28S rRNA gene were amplified.

Supplementary table S3. Conditions of PCR reactions used for amplification of five molecular markers analyzed in astome ciliates and their earthworm hosts.

Molecular marker	Organism group	PCR program			Reference
		**Initial denaturation**	**Cycling (denaturation, annealing, extension)**	**Final extension**	
18S rRNA gene	Plagiotomids	95 °C/15 min	30 cycles: 95 °C/45 s, 55 °C/60 s, 72 °C/150 s	72 °C/10 min	Vďačný et al. (2011)
ITS region and 28S rRNA gene[a]	Plagiotomids	95 °C/15 min	35 cycles: 95 °C/45 s, 55 °C/60 s, 72 °C/150 s	72 °C/10 min	Vďačný et al. (2011)[b]
Cytochrome *c* oxidase subunit I (COI)	Plagiotomids	94 °C/4 min	5 cycles: 94 °C/45 s, 45 °C/75 s, 72 °C/90 s<br>35 cycles: 94 °C/45 s, 55 °C/75 s, 72 °C/90 s	72 °C/8 min	Rataj and Vďačný (2020)
	Earthworms	95 °C/5 min	40 cycles: 95 °C/30 s, 50 °C/90s, 72 °C/180 s	72 °C/10 min	Kolicka (2019)
NADH-ubiquinone oxidoreductase chain 1 (ND1)	Earthworms	94 °C/4 min	5 cycles: 94 °C/45 s, 45 °C/75 s, 72 °C/90 s<br>35 cycles: 94 °C/45 s, 55 °C/75 s, 72 °C/90 s	72 °C/8 min	Rataj and Vďačný (2020)

[a] The barcoding D1/D2 domains of the 28S rRNA gene were amplified.

[b] Modified from Vďačný et al. (2011) by adding five cycles.

Supplementary table S4. Morphometric data on 52 specimens isolated from three different earthworm species.

Host species	Specimen	V1	V2	V3	V4	V5	V6	V7	V8	V9	V10	V11	V12	V13	V14	V15	V16	V17	V18	V19	V20	V21
Aporrectodea tuburculata	AT1	243.1	108.8	2.2	66.2	145.1	16.0	6.8	2.4	20.3	8.0	2.5	14	5.2	22	23	158.8	65.3	131.9	39.8	75	
	AT2	220.7	100.8	2.2	59.3	140.3	14.0	8.8	1.6	7.1	3.4	2.1	19	4.7	23	24	140.8	63.8	123.8	35.4	72	2.0
	AT3	218.3	86.3	2.5	63.7	133.7	15.5	8.0	1.9	21.0	5.9	3.6	18	4.9	24	23	145.4	66.6	127.8	33.3	76	1.7
	AT4	192.4	90.9	2.1	56.5	126.8	11.1	5.6	2.0	10.1	6.7	1.5	19	6.6	21	21	122.1	63.5	110.3	30.5	71	
	AT5	174.2	69.4	2.5	52.8	115.1	18.9	9.8	1.9	19.0	7.4	2.6	17	5.8	18	21	117.2	67.3	98.2	26.2	69	
	AT6	193.9	67.8	2.9	55.2	116.9	6.2	3.6	1.7	11.2	4.5	2.5	19	4.5	20	23	117.6	60.6	98.5	23.1	77	2.4
	AT7	164.7	58.7	2.8	41.5	105.8	16.6	5.0	3.3	11.7	5.7	2.1	19	4.5	23	22	110.5	67.1	92.3	22.6	75	
	AT8	204.8	80.8	2.5	67.7	113.4	8.7	5.4	1.6	6.4	4.3	1.5	20		24	23	116.8	57.0	96.3	22.6	73	
	AT9	188.7	75.8	2.5	55.8	111.9	12.9	5.8	2.2	11.7	4.5	2.6	19	5.1	20	24	112.7	59.7	92.6	23.7	73	
	AT10	171.4	61.8	2.8	53.4	98.5	6.2	3.8	1.6	12.1	4.7	2.6	23	5.1	23	23	105.4	61.5	87.6	20.2	71	2.0
	AT11	191.7	74.1	2.6	60.9	109.7	14.3	7.5	1.9	12.5	6.3	2.0	19	7.3	25	25	114.7	59.8	94.7	23.5	76	1.7
	AT12	190.0	73.0	2.6	59.0	108.8	14.7	7.1	2.1	12.0	5.0	2.4	20	4.5	20	23	112.0	58.9	92.8	22.9	74	2.1
	AT13	171.2	71.3	2.4	48.3	105.2	11.0	6.3	1.7	14.0	6.8	2.1	18	5.7	22	22	106.2	62.0	89.2	20.5	74	1.8
	AT14	194.2	78.4	2.5	54.2	68.4	7.7	3.0	2.6	10.0	4.0	2.5	19	4.1	22	22	100.1	51.5	85.2	21.1	71	2.2
	AT15	164.4	70.5	2.3	49.8	99.0	11.2	4.2	2.7	7.3	2.7	2.7	23	4.7	21	20	97.3	59.2	87.1	20.2	73	
	AT16	185.5	65.4	2.8	59.6	106.0	11.9	6.7	1.8	8.5	5.5	1.5	17	6.0	24	25	115.4	62.2	97.5	25.6	77	2.0
	AT17	182.3	66.6	2.7	54.4	108.4	15.9	8.8	1.8	15.4	6.0	2.6	18	6.0	22	21	110.4	60.6	91.1	23.4	76	2.2
	AT18	191.3	58.2	3.3	51.3	98.4	14.0	5.5	2.5	8.0	4.2	1.9	19	5.0	25	26	105.9	55.4	91.1	21.8	77	1.8
	AT19	188.3	75.2	2.5	54.5	115.4	14.5	6.8	2.1	13.9	5.3	2.6	19	4.6	23	24	107.7	57.2	90.5	22.6	72	1.9
	AT20	169.8	68.2	2.5	55.8	100.0	11.4	5.5	2.1	10.4	4.6	2.3	18	4.6	24	24	99.2	58.4	81.2	20.6	66	
	AT21	165.7	68.3	2.4	53.2	97.8	12.8	5.3	2.4	7.7	4.8	1.6	19	5.2	20	22	98.3	59.3	86.8	19.4	65	
Lumbricus terrestris	LT1	146.0	46.0	3.2	41.6	92.5	6.6	5.1	1.3	4.7	5.0	0.9	15	5.0	21	20	94.7	64.9	79.1	21.6	66	2.0
	LT2	167.0	54.0	3.1	53.0	104.0	12.6	7.9	1.6	7.9	5.7	1.4	17	5.8	18	23	107.1	64.1	81.0	107.0	64	
	LT3	148.0	50.0	3.0	44.0	92.8	7.0	4.5	1.6	10.0	5.4	1.9	19	5.0	16	22	98.0	66.2	81.0	17.0	64	1.5
	LT4	168.0	57.0	2.9	49.0	103.7	14.3	5.6	2.6	14.1	5.1	2.8	16	5.1	17	22	103.5	61.6	84.7	20.8	72	
	LT5	173.0	60.0	2.9	47.5	107.5	7.6	5.0	1.5	8.5	5.3	1.6	18	5.1	18	21	100.5	58.1	85.8	20.0	68	2.0
	LT6	172.0	60.0	2.9	51.5	104.5	8.2	5.3	1.5	6.3	4.1	1.5	21	5.3	18	23	103.9	60.4	87.7	22.0	67	
	LT7	146.7	48.0	3.1	40.5	92.9	4.5	5.0	0.9	7.0	4.0	1.8	19	4.3	19	25	95.5	65.1	77.6	18.0	64	

	LT8	140.0	46.0	3.0	38.5	84.5	8.9	3.4	2.6	9.1	4.0	2.3	19	5.3	20	21	88.5	63.2	73.4	16.0	65	1.8
	LT9	135.0	44.4	3.0	40.0	81.4	9.0	5.3	1.7	11.0	4.1	2.7	16	5.3	19	21	89.8	66.5	76.0	20.0	66	2.0
	LT10	136.0	42.0	3.2	38.7	85.5	9.2	4.2	2.2	8.0	4.2	1.9	18	4.4	20	22	91.0	66.9	77.0	19.8	67	1.9
	LT11	140.0	44.5	3.1	39.6	88.0	7.7	5.2	1.5	4.6	3.8	1.2	22	5.0	19	22	94.8	67.7	77.8	20.7	67	2.0
	LT12	155.7	50.4	3.1	39.8	102.7	5.6	4.5	1.2	7.6	4.8	1.6	21	4.9	17	21	105.2	67.6	85.6	22.0	68	1.6
	LT13	175.0	59.2	3.0	48.9	106.5	12.7	5.5	2.3	9.1	4.8	1.9	19	6.8	19	21	110.6	63.2	91.0	21.0	68	2.0
	LT14	157.4	57.5	2.7	47.3	97.4	8.8	5.1	1.7	11.0	4.3	2.6	18	4.3	18	22	98.0	62.3	81.7	20.7	63	1.9
	LT15	145.3	53.5	2.7	44.5	84.9	6.3	6.4	1.0	13.3	8.4	1.6	21	5.4	17	22	103.1	71.0	85.7	20.3	64	1.7
	LT16	179.7	71.9	2.5	50.0	113.2	8.7	5.1	1.7	14.0	6.0	2.3	17	5.5	18	22	105.0	58.4	82.5	21.7	64	
	LT17	153.9	58.8	2.6	41.2	99.6	15.8	7.0	2.3	7.6	2.8	2.7	19	5.7	19	23	94.9	61.7	77.9	20.3	61	1.6
	LT18	183.5	65.5	2.8	48.3	111.3	11.7	5.4	2.2	8.6	4.0	2.2	20	4.7	20	21	106.8	58.2	89.3	22.9	69	2.0
Lumbricus rubellus	LR1	161.3	65.0	2.5	41.0	82.5	5.8	4.3	1.3	7.1	4.4	1.6	17	5.3	18	19	95.6	59.3	75.2	21.5	59	
	LR2	136.2	44.7	3.0	28.8	87.8	11.7	5.0	2.3	11.3	4.5	2.5	19	6.9	16	18	87.8	64.5	66.4	19.8	59	2.0
	LR3	148.4	56.2	2.6	37.2	72.4	7.0	4.7	1.5	6.3	4.0	1.6	15	6.3	15	22	88.9	59.9	67.4	20.6	55	
	LR4	160.5	62.5	2.6	36.3	95.2	9.4	4.8	2.0	12.0	5.6	2.1	18	6.3	17	20	90.8	56.6	67.5	22.3	58	2.0
	LR5	113.0	47.2	2.4	23.0	67.8	6.9	2.6	2.7	7.7	4.7	1.6	15	4.8	16	23	73.0	64.6	50.3	21.9	51	2.1
	LR6	129.6	51.5	2.5	23.9	75.9	9.9	3.8	2.6	11.1	3.0	3.7	15	5.6	17	24	77.6	59.9	56.4	22.7	55	2.2
	LR7	122.0	47.1	2.6	29.0	55.0	8.8	3.6	2.4	9.5	3.0	3.2	18	5.5	16	24	75.2	61.6	57.0	18.6	51	1.7
	LR8	124.8	50.3	2.5	22.2	70.8	7.2	3.2	2.3	7.5	4.3	1.7	18	4.7	16	21	76.4	61.2	56.7	24.3	55	1.7
	LR9	137.0	47.0	2.9	28.1	80.0	6.5	5.0	1.3	11.1	3.4	3.3	19	5.2	15	20	80.1	58.5	63.4	17.5	56	2.1
	LR10	120.0	44.0	2.7	23.7	61.4	7.3	4.0	1.8	10.0	4.0	2.5	14	5.7	17	23	74.4	62.0	56.4	19.0	54	
	LR11	156.3	64.3	2.4	30.3	90.6	12.5	6.6	1.9	13.7	4.1	3.3	16	7.2	18	25	87.2	55.8	68.4	18.4	60	2.4
	LR12	142.4	49.1	2.9	27.0	90.0	10.2	4.6	2.2	10.8	3.4	3.2	22	6.3	15	23	83.7	58.8	61.5	20.0	57	
	LR13	155.0	57.1	2.7	29.5	93.6	6.6	4.0	1.7	10.6	4.7	2.3	24	6.7	17	22	92.3	59.5	69.0	20.3	61	2.2

V1, body length; V2, body width; V3, body length:width ratio; V4, distance of anterior body end to anteriormost macronuclear nodule; V5, length of nuclear figure; V6, length of anteriormost macronuclear nodule; V7, width of anteriormost macronuclear nodule; V8, anteriormost macronuclear nodule, length:width ratio; V9, length of posteriormost macronuclear nodule; V10, width of posteriormost macronuclear nodule; V11, posteriormost macronuclear nodule length:width ratio; V12, number of macronuclear nodules; V13, largest diameter of micronucleus; V14, number of ventral cirral rows; V15, number of dorsal cirral rows; V16, distance of anterior body end to proximal end of adoral zone of membranelles (AZM); V17, body length:AZM length ratio (%); V18, length of straight portion of AZM; V19, length of bent portion of AZM; V20, number of adoral membranelles; V21, length of paroral membrane.

Supplementary table S5. Characterization and origin of nuclear and mitochondrial gene sequences of plagiotomids analyzed in the present study.

Taxon	Specimen[a]	Host species	Locality code[b]	18S rRNA gene	ITS1-5.8S-ITS2-28S rRNA gene[c]	Cytochrome *c* oxidase subunit I
Plagiotoma lumbrici complex	RZ 1-LT	*Lumbricus terrestris*	RZ	OP538845	OP538960	OP562267
Plagiotoma lumbrici complex	RZ 2-LT	*Lumbricus terrestris*	RZ	OP538846	OP538961	OP562268
Plagiotoma lumbrici complex	RZ 3-LT	*Lumbricus terrestris*	RZ	OP538847	OP538962	OP562269
Plagiotoma lumbrici complex	KR 7-LT	*Lumbricus terrestris*	KR	OP538848	OP538963	OP562270
Plagiotoma lumbrici complex	JA-2 6/23-LT	*Lumbricus terrestris*	JA-2	OP538849	OP538964	OP562271
Plagiotoma lumbrici complex	JA-2 6/24-LT	*Lumbricus terrestris*	JA-2	OP538850	OP538965	OP562272
Plagiotoma lumbrici complex	PUz 38 LT	*Lumbricus terrestris*	PUz	OP538851	OP538966	OP562273
Plagiotoma lumbrici complex	PUz 39 LT	*Lumbricus terrestris*	PUz	OP538852	OP538967	OP562274
Plagiotoma lumbrici complex	KD 42 LT	*Lumbricus terrestris*	KD	OP538853	OP538968	OP562275
Plagiotoma lumbrici complex	SL 61 LT	*Lumbricus terrestris*	SL	OP538854	OP538969	OP562276
Plagiotoma lumbrici complex	SL 62 LT	*Lumbricus terrestris*	SL	OP538855	OP538970	OP562277
Plagiotoma lumbrici complex	SL 63 LT	*Lumbricus terrestris*	SL	OP538856	OP538971	OP562278
Plagiotoma lumbrici complex	PUh 67 LR	*Lumbricus rubellus*	PUh	OP538857	OP538972	OP562279
Plagiotoma lumbrici complex	PUh 68 LR	*Lumbricus rubellus*	PUh	OP538858	OP538973	OP562280
Plagiotoma lumbrici complex	PUh 69 LR	*Lumbricus rubellus*	PUh	OP538859	OP538974	OP562281
Plagiotoma lumbrici complex	PUh 70 LR	*Lumbricus rubellus*	PUh	OP538860	OP538975	OP562282
Plagiotoma lumbrici complex	FNS 71 LT	*Lumbricus terrestris*	FNS	OP538861	OP538976	OP562283
Plagiotoma lumbrici complex	FNS 72 LT	*Lumbricus terrestris*	FNS	OP538862	OP538977	OP562284
Plagiotoma lumbrici complex	PUh 92 LR	*Lumbricus rubellus*	PUh	OP538863	OP538978	OP562285
Plagiotoma lumbrici complex	PUh 93 LR	*Lumbricus rubellus*	PUh	OP538864	OP538979	OP562286
Plagiotoma lumbrici complex	PUh 94 LR	*Lumbricus rubellus*	PUh	OP538865	OP538980	OP562287
Plagiotoma lumbrici complex	PUh 95 LR	*Lumbricus rubellus*	PUh	OP538866	OP538981	OP562288
Plagiotoma lumbrici complex	PUh 96 LR	*Lumbricus rubellus*	PUh	OP538867	OP538982	OP562289
Plagiotoma lumbrici complex	PUh 97 LR	*Lumbricus rubellus*	PUh	OP538868	OP538983	OP562290
Plagiotoma lumbrici complex	TT 99 LT	*Lumbricus terrestris*	TT	OP538869	OP538984	OP562291
Plagiotoma lumbrici complex	TT 100 LT	*Lumbricus terrestris*	TT	OP538870	OP538985	OP562292
Plagiotoma lumbrici complex	TT 101 LT	*Lumbricus terrestris*	TT	OP538871	OP538986	OP562293
Plagiotoma lumbrici complex	TT 102 LT	*Lumbricus terrestris*	TT	OP538872	OP538987	OP562294
Plagiotoma lumbrici complex	JA 112 LT	*Lumbricus terrestris*	JA-3	OP538873	OP538988	OP562295

Plagiotoma lumbrici complex	JA 113 LT	*Lumbricus terrestris*	JA-3	OP538874	OP538989	OP562296
Plagiotoma lumbrici complex	PUh 117 LT	*Lumbricus terrestris*	PUh	OP538875	OP538990	OP562297
Plagiotoma lumbrici complex	PUh 118 LT	*Lumbricus terrestris*	PUh	OP538876	OP538991	OP562298
Plagiotoma lumbrici complex	PUh 119 LT	*Lumbricus terrestris*	PUh	OP538877	OP538992	OP562299
Plagiotoma lumbrici complex	PUh 120 LT	*Lumbricus terrestris*	PUh	OP538878	OP538993	OP562300
Plagiotoma lumbrici complex	PUh 121 LT	*Lumbricus terrestris*	PUh	OP538879	OP538994	OP562301
Plagiotoma lumbrici complex	PUh 122 LT	*Lumbricus terrestris*	PUh	OP538880	OP538995	OP562302
Plagiotoma lumbrici complex	PUh 124 LT	*Lumbricus terrestris*	PUh	OP538881	OP538996	OP562303
Plagiotoma lumbrici complex	PUh 125 LT	*Lumbricus terrestris*	PUh	OP538882	OP538997	OP562304
Plagiotoma lumbrici complex	PUh 126 LT	*Lumbricus terrestris*	PUh	OP538883	OP538998	OP562305
Plagiotoma lumbrici complex	PUh 127 LR	*Lumbricus rubellus*	PUh	OP538884	OP538999	OP562306
Plagiotoma lumbrici complex	PUh 128 LR	*Lumbricus rubellus*	PUh	OP538885	OP539000	OP562307
Plagiotoma lumbrici complex	PUh 129 LR	*Lumbricus rubellus*	PUh	OP538886	OP539001	OP562308
Plagiotoma lumbrici complex	PUh 130 LR	*Lumbricus rubellus*	PUh	OP538887	OP539002	OP562309
Plagiotoma lumbrici complex	PUh 131 LR	*Lumbricus rubellus*	PUh	OP538888	OP539003	OP562310
Plagiotoma lumbrici complex	PUh 132 LR	*Lumbricus rubellus*	PUh	OP538889	OP539004	OP562311
Plagiotoma lumbrici complex	PUh 133 LR	*Lumbricus rubellus*	PUh	OP538890	OP539005	OP562312
Plagiotoma lumbrici complex	PUh 134 LR	*Lumbricus rubellus*	PUh	OP538891	OP539006	OP562313
Plagiotoma lumbrici complex	PUh 135 LR	*Lumbricus rubellus*	PUh	OP538892	OP539007	OP562314
Plagiotoma lumbrici complex	PUh 136 LR	*Lumbricus rubellus*	PUh	OP538893	OP539008	OP562315
Plagiotoma lumbrici complex	PUh 137 LR	*Lumbricus rubellus*	PUh	OP538894	OP539009	OP562316
Plagiotoma lumbrici complex	PUh 138 LR	*Lumbricus rubellus*	PUh	OP538895	OP539010	OP562317
Plagiotoma lumbrici complex	PUh 139 LR	*Lumbricus rubellus*	PUh	OP538896	OP539011	OP562318
Plagiotoma lumbrici complex	PUh 140 LR	*Lumbricus rubellus*	PUh	OP538897	OP539012	OP562319
Plagiotoma lumbrici complex	PUh 141 LR	*Lumbricus rubellus*	PUh	OP538898	OP539013	OP562320
Plagiotoma lumbrici complex	PUh 142 LR	*Lumbricus rubellus*	PUh	OP538899	OP539014	OP562321
Plagiotoma lumbrici complex	JV 159 LT	*Lumbricus terrestris*	JV	OP538900	OP539015	OP562322
Plagiotoma lumbrici complex	JV 160 LT	*Lumbricus terrestris*	JV	OP538901	OP539016	OP562323
Plagiotoma lumbrici complex	JV 161 LT	*Lumbricus terrestris*	JV	OP538902	OP539017	OP562324
Plagiotoma lumbrici complex	JV 162 LT	*Lumbricus terrestris*	JV	OP538903	OP539018	OP562325
Plagiotoma lumbrici complex	JV 163 LT	*Lumbricus terrestris*	JV	OP538904	OP539019	OP562326
Plagiotoma lumbrici complex	JV 164 LT	*Lumbricus terrestris*	JV	OP538905	OP539020	OP562327
Plagiotoma lumbrici complex	JV 165 LT	*Lumbricus terrestris*	JV	OP538906	OP539021	OP562328

Plagiotoma lumbrici complex	JV 166 LT	*Lumbricus terrestris*	JV	OP538907	OP539022	OP562329
Plagiotoma lumbrici complex	JV 167 LT	*Lumbricus terrestris*	JV	OP538908	OP539023	OP562330
Plagiotoma lumbrici complex	JV 168 LT	*Lumbricus terrestris*	JV	OP538909	OP539024	OP562331
Plagiotoma lumbrici complex	PUh 212 LR	*Lumbricus rubellus*	PUh	OP538910	OP539025	OP562332
Plagiotoma lumbrici complex	PUh 213 LR	*Lumbricus rubellus*	PUh	OP538911	OP539026	OP562333
Plagiotoma lumbrici complex	PUh 214 LR	*Lumbricus rubellus*	PUh	OP538912	OP539027	OP562334
Plagiotoma lumbrici complex	PUh 215 LR	*Lumbricus rubellus*	PUh	OP538913	OP539028	OP562335
Plagiotoma lumbrici complex	PUh 216 LR	*Lumbricus rubellus*	PUh	OP538914	OP539029	OP562336
Plagiotoma lumbrici complex	PUh 217 LR	*Lumbricus rubellus*	PUh	OP538915	OP539030	OP562337
Plagiotoma lumbrici complex	PUh 218 LR	*Lumbricus rubellus*	PUh	OP538916	OP539031	OP562338
Plagiotoma lumbrici complex	PUh 219 LR	*Lumbricus rubellus*	PUh	OP538917	OP539032	OP562339
Plagiotoma lumbrici complex	PUh 220 LR	*Lumbricus rubellus*	PUh	OP538918	OP539033	OP562340
Plagiotoma lumbrici complex	PUh 221 LR	*Lumbricus rubellus*	PUh	OP538919	OP539034	OP562341
Plagiotoma lumbrici complex	PUh 222 LR	*Lumbricus rubellus*	PUh	OP538920	OP539035	OP562342
Plagiotoma lumbrici complex	PUh 223 LR	*Lumbricus rubellus*	PUh	OP538921	OP539036	OP562343
Plagiotoma lumbrici complex	PUh 224 LR	*Lumbricus rubellus*	PUh	OP538922	OP539037	OP562344
Plagiotoma lumbrici complex	PUh 225 LR	*Lumbricus rubellus*	PUh	OP538923	OP539038	OP562345
Plagiotoma aporrectodeae	LS 43 AT	*Aporrectodea tuberculata*	LS	OP538924	OP539039	OP562346
Plagiotoma aporrectodeae	LS 44 AT	*Aporrectodea tuberculata*	LS	OP538925	OP539040	OP562347
Plagiotoma aporrectodeae	LS 45 AT	*Aporrectodea tuberculata*	LS	OP538926	OP539041	OP562348
Plagiotoma aporrectodeae	LS 46 AT	*Aporrectodea tuberculata*	LS	OP538927	OP539042	OP562349
Plagiotoma aporrectodeae	LS 48 AT	*Aporrectodea tuberculata*	LS	OP538928	OP539043	OP562350
Plagiotoma aporrectodeae	LS 49 AT	*Aporrectodea tuberculata*	LS	OP538929	OP539044	OP562351
Plagiotoma aporrectodeae	AMc 50 AT	*Aporrectodea tuberculata*	AMc	OP538930	OP539045	OP562352
Plagiotoma aporrectodeae	AMc 51 AT	*Aporrectodea tuberculata*	AMc	OP538931	OP539046	OP562353
Plagiotoma aporrectodeae	HkD 53 AT	*Aporrectodea tuberculata*	HkD	OP538932	OP539047	OP562354
Plagiotoma aporrectodeae	HkD 54 AT	*Aporrectodea tuberculata*	HkD	OP538933	OP539048	OP562355
Plagiotoma aporrectodeae	HkD 55 AT	*Aporrectodea tuberculata*	HkD	OP538934	OP539049	OP562356
Plagiotoma aporrectodeae	PUnp 64 AT	*Aporrectodea tuberculata*	PUnp	OP538935	OP539050	OP562357
Plagiotoma aporrectodeae	PUnp 65 AT	*Aporrectodea tuberculata*	PUnp	OP538936	OP539051	OP562358
Plagiotoma aporrectodeae	RZ 103 AT	*Aporrectodea tuberculata*	RZ	OP538937	OP539052	OP562359
Plagiotoma aporrectodeae	RZ 105 AT	*Aporrectodea tuberculata*	RZ	OP538938	OP539053	OP562360
Plagiotoma aporrectodeae	RZ 107 AT	*Aporrectodea tuberculata*	RZ	OP538939	OP539054	OP562361

Plagiotoma aporrectodeae	RZ 108 AT	*Aporrectodea tuberculata*	RZ	OP538940	OP539055	OP562362
Plagiotoma aporrectodeae	RZ 109 AT	*Aporrectodea tuberculata*	RZ	OP538941	OP539056	OP562363
Plagiotoma aporrectodeae	RZ 110 AT	*Aporrectodea tuberculata*	RZ	OP538942	OP539057	OP562364
Plagiotoma aporrectodeae	RZ 111 AT	*Aporrectodea tuberculata*	RZ	OP538943	OP539058	OP562365
Plagiotoma aporrectodeae	JV 143 AT	*Aporrectodea tuberculata*	JV	OP538944	OP539059	OP562366
Plagiotoma aporrectodeae	JV 144 AT	*Aporrectodea tuberculata*	JV	OP538945	OP539060	OP562367
Plagiotoma aporrectodeae	JV 145 AT	*Aporrectodea tuberculata*	JV	OP538946	OP539061	OP562368
Plagiotoma aporrectodeae	JV 146 AT	*Aporrectodea tuberculata*	JV	OP538947	OP539062	OP562369
Plagiotoma aporrectodeae	JV 147 AT	*Aporrectodea tuberculata*	JV	OP538948	OP539063	OP562370
Plagiotoma aporrectodeae	JV 148 AT	*Aporrectodea tuberculata*	JV	OP538949	OP539064	OP562371
Plagiotoma aporrectodeae	JV 149 AT	*Aporrectodea tuberculata*	JV	OP538950	OP539065	OP562372
Plagiotoma aporrectodeae	JV 150 AT	*Aporrectodea tuberculata*	JV	OP538951	OP539066	OP562373
Plagiotoma aporrectodeae	JV 151 AT	*Aporrectodea tuberculata*	JV	OP538952	OP539067	OP562374
Plagiotoma aporrectodeae	JV 152 AT	*Aporrectodea tuberculata*	JV	OP538953	OP539068	OP562375
Plagiotoma aporrectodeae	JV 153 AT	*Aporrectodea tuberculata*	JV	OP538954	OP539069	OP562376
Plagiotoma aporrectodeae	JV 154 AT	*Aporrectodea tuberculata*	JV	OP538955	OP539070	OP562377
Plagiotoma aporrectodeae	JV 155 AT	*Aporrectodea tuberculata*	JV	OP538956	OP539071	OP562378
Plagiotoma aporrectodeae	JV 156 AT	*Aporrectodea tuberculata*	JV	OP538957	OP539072	OP562379
Plagiotoma aporrectodeae	JV 157 AT	*Aporrectodea tuberculata*	JV	OP538958	OP539073	OP562380
Plagiotoma aporrectodeae	JV 158 AT	*Aporrectodea tuberculata*	JV	OP538959	OP539074	OP562381

[a] Specimen code consists of a locality code as specified in Supplementary Table S1, an isolate code, and an abbreviation of host species name (AT, *Aporrectodea tuberculata*; LR, *Lumbricus rubellus*; LT, *Lumbricus terrestris*).

[b] For locality codes and further details, see Supplementary Table S1.

[c] The 28S rRNA gene includes here only the first two barcoding domains.

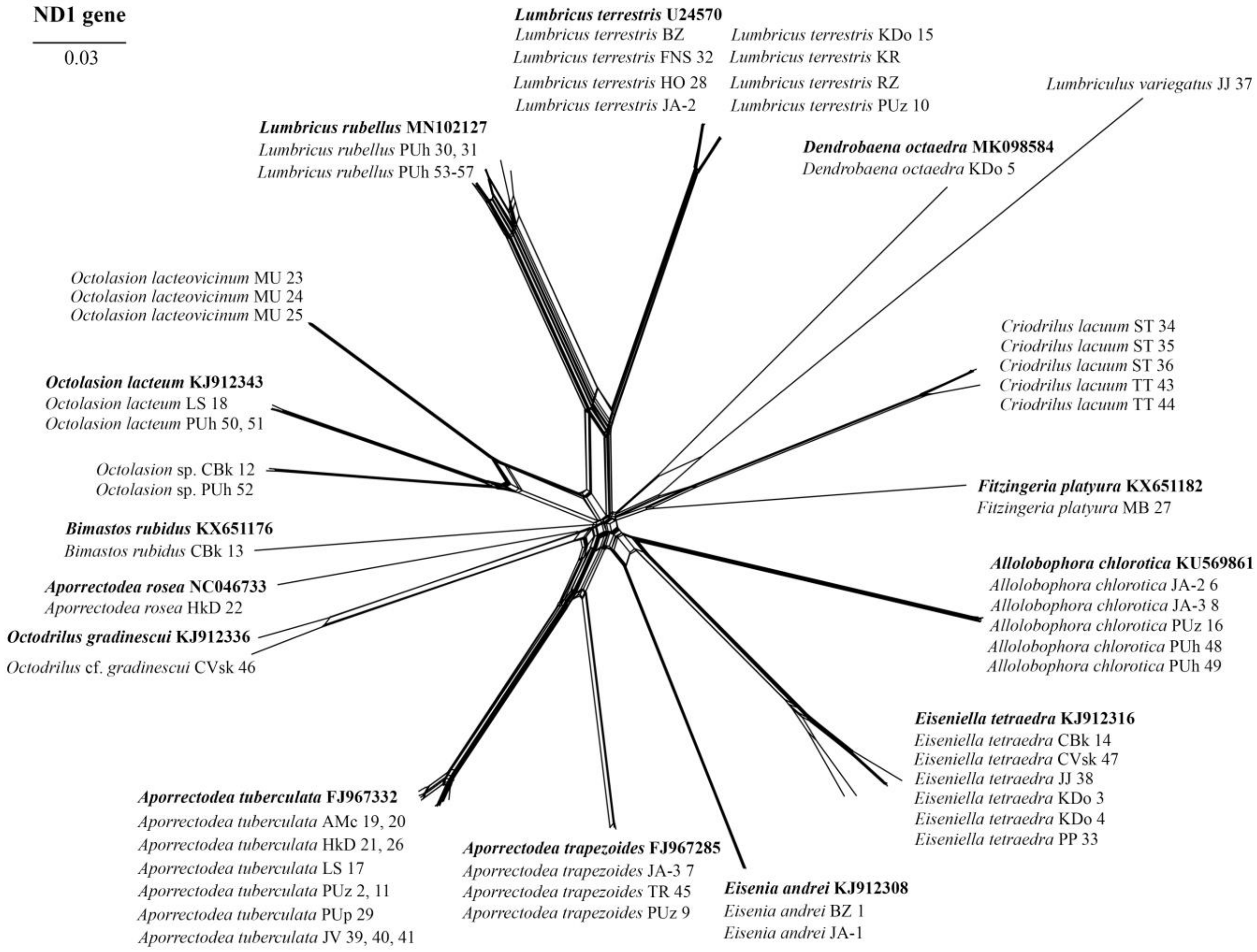

Supplementary Fig. S1. Phylogenetic network computed from NADH-ubiquinone oxidoreductase chain 1 (ND1) sequences of the studied lumbricid earthworms, using the neighbornet algorithm and the uncorrected distances in SplitsTree ver. 4. Sequences in bold face were downloaded from GenBank and served for the classification of the earthworm species studied. The scale bar indicates three substitutions per one hundred nucleotide positions.

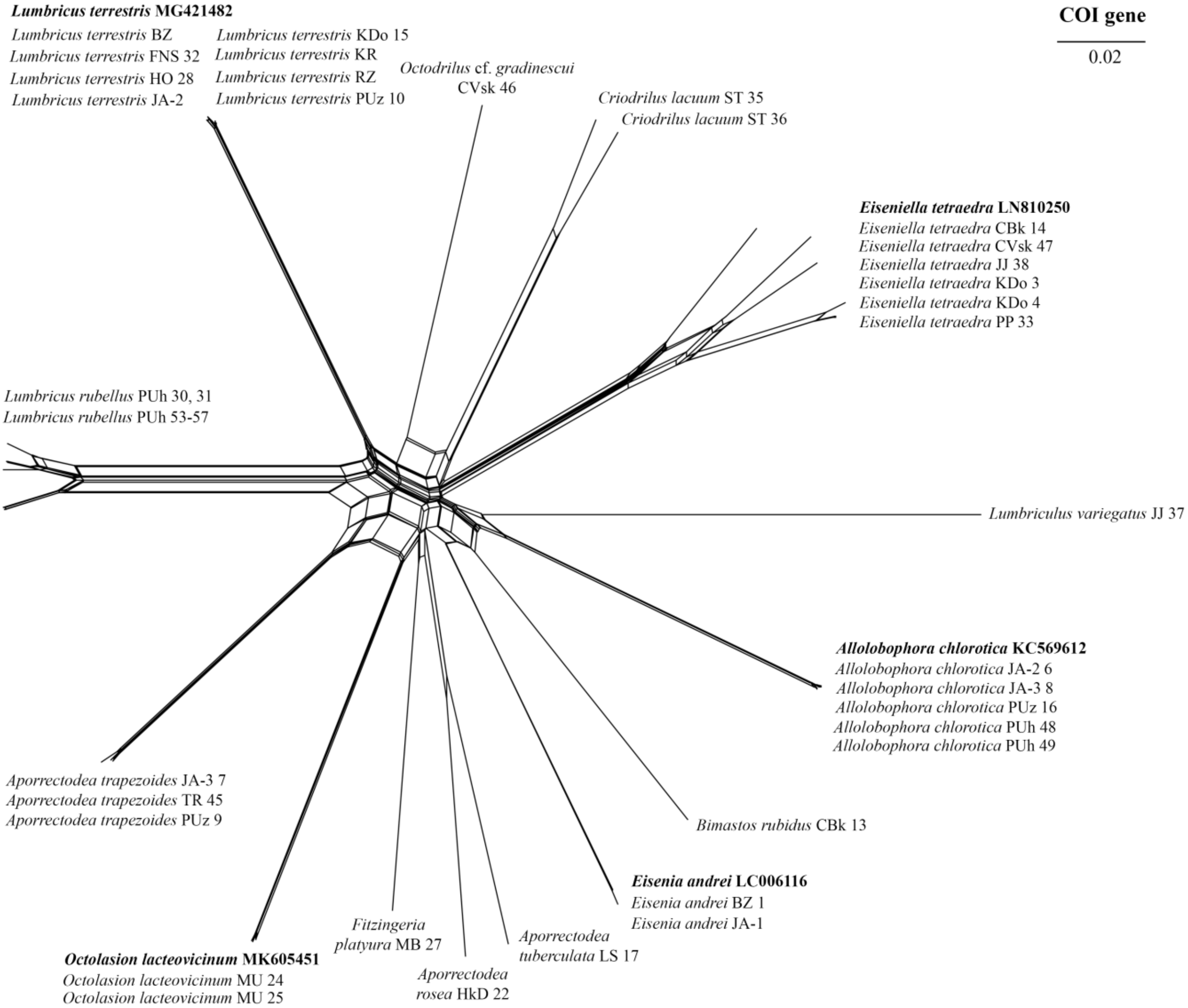

Supplementary Fig. S2. Phylogenetic network computed from cytochrome *c* oxidase subunit I (COI) sequences of lumbricid earthworms, using the neighbornet algorithm and the uncorrected distances in SplitsTree ver. 4. Sequences in bold face were downloaded from GenBank and served for the classification of the earthworm species carrying astome ciliates. The scale bar indicates three substitutions per one hundred nucleotide positions.

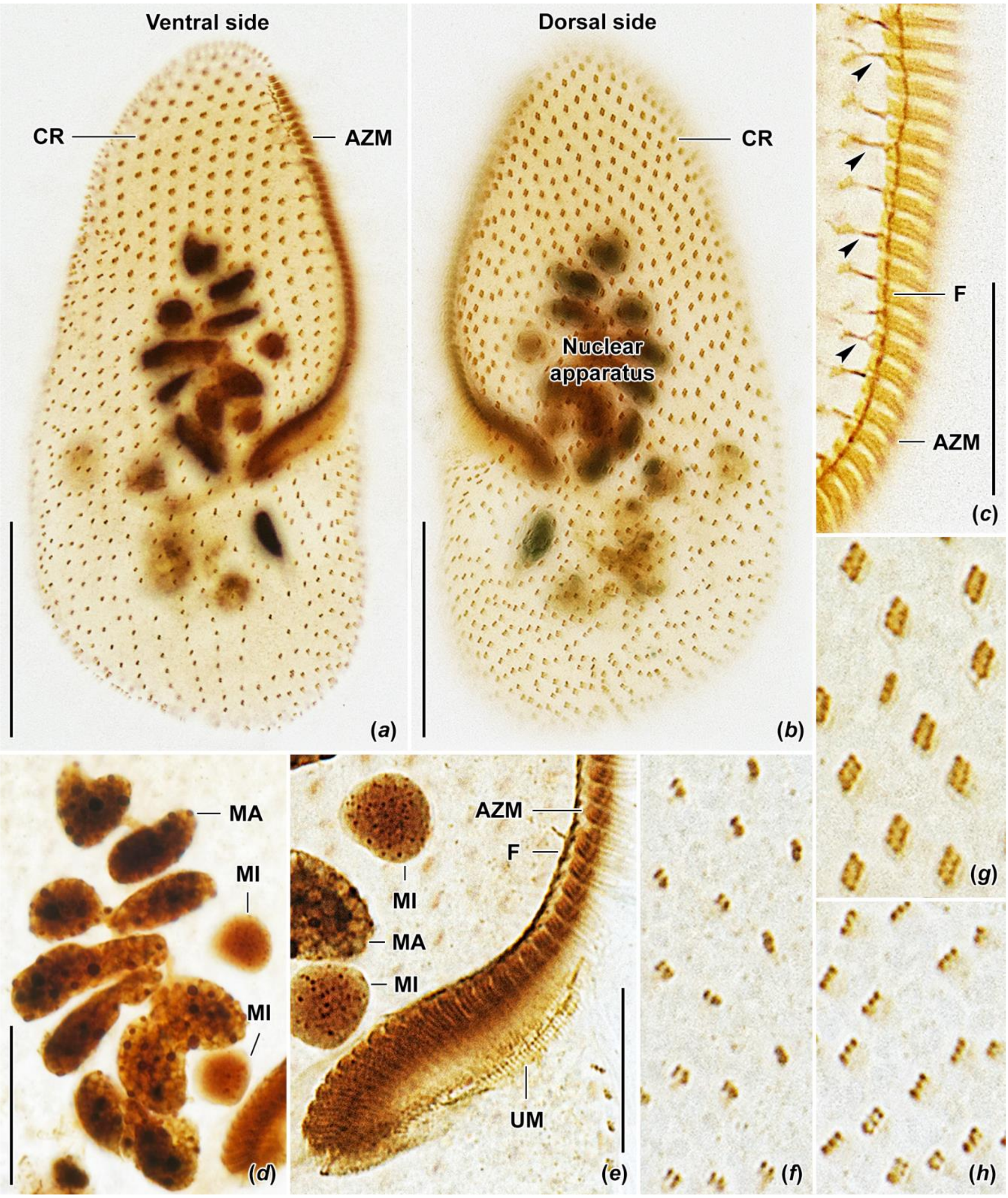

Supplementary Fig. S3. *Plagiotoma lumbrici* complex, isolated from *Lumbricus terrestris* after protargol impregnation. (*a, b*) Cirral pattern of ventral and dorsal sides. (*c*) Detail showing the fine structure of the adoral zone of membranelles. Arrowheads denote fibres connecting cirri with the submembranellar fibre bundle. (*d*) The nuclear apparatus consists of a branched macronuclear strand and two globular micronuclei. (*e*) Detail showing the fine structure of the proximal region of the adoral zone of membranelles. (*f*–*h*) Fine structure of cirri in the anterior body region (*g*), mid-body (*h*), and posterior body region (*h*. AZM, adoral zone of membranelles; CR, cirral rows; F, submembranellar fibre bundle; MA, macronucleus; MI, micronuclei; UM, undulating membranes. Scale bars: 10 µm (*c, e*), 20 µm (*d*), and 50 µm (*a, b*).

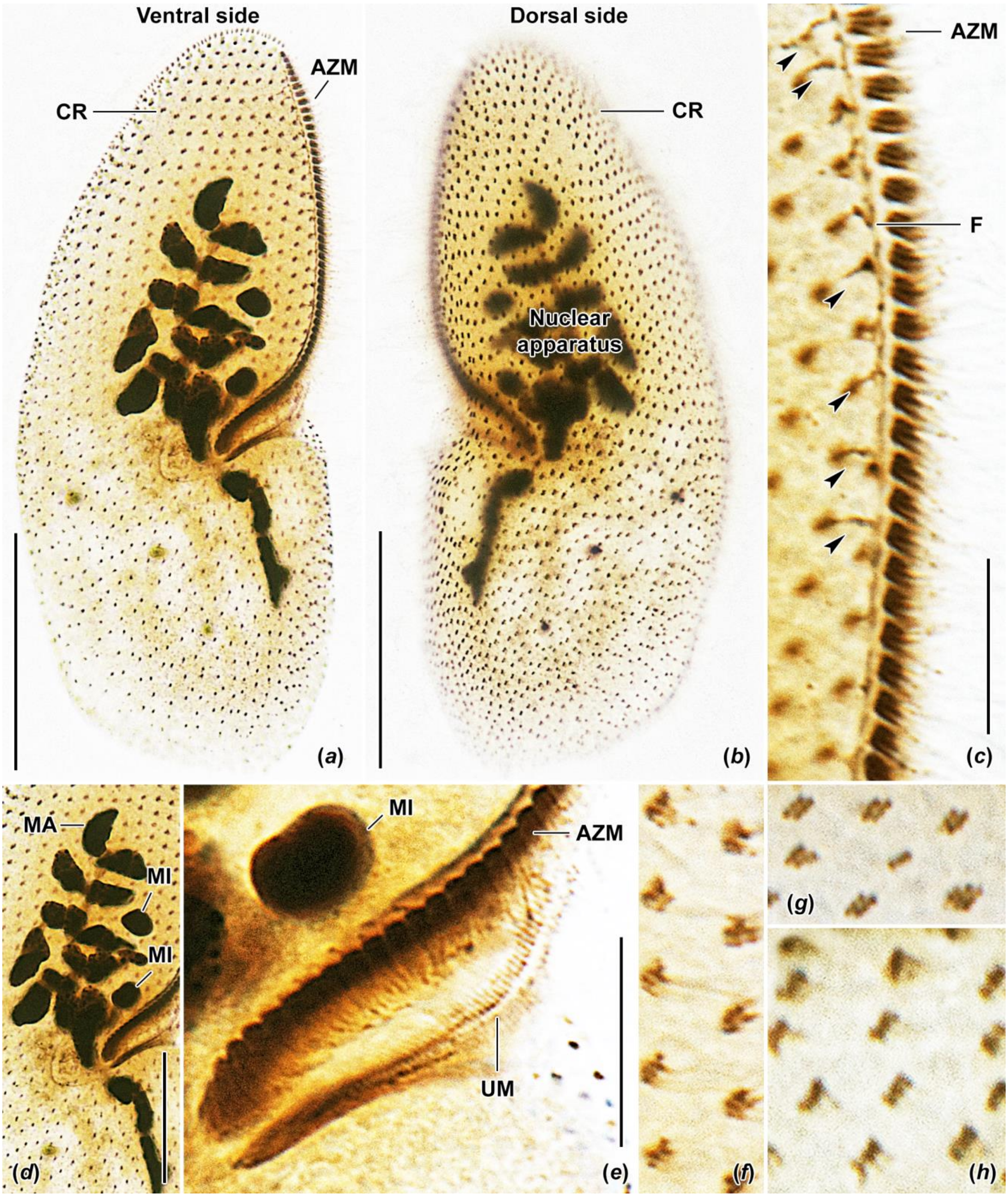

Supplementary Fig. S4. *Plagiotoma lumbrici* complex, isolated from *Lumbricus rubellus* after protargol impregnation. (*a, b*) Cirral pattern of ventral and dorsal sides. (*c*) Detail showing the fine structure of the adoral zone of membranelles. Arrowheads denote fibres connecting cirri with the submembranellar fibre bundle. (*d*) The nuclear apparatus consists of a branched macronuclear strand and two globular micronuclei. (*e*) Detail showing the fine structure of the proximal region of the adoral zone of membranelles. (*f*–*h*) Fine structure of cirri in the anterior body region (*f*), mid-body (*g*), and posterior body region (*h*). AZM, adoral zone of membranelles; CR, cirral rows; F, submembranellar fibre bundle; MA, macronucleus; MI, micronuclei; UM, undulating membranes. Scale bars: 10 µm (*c, e*), 20 µm (*d*), and 50 µm (*a, b*).

References

Folmer, O., Black, M., Hoeh, W., Lutz, R., and Vrijenhoek, R. (1994). DNA primers for amplification of mitochondrial cytochrome *c* oxidase subunit I from diverse metazoan invertebrates. *Mol. Mar. Biol. Biotechnol.* 3, 294–299.

Kolicka, M. (2019). New *Chaetonotus* (*Wolterecka*) *semovitus* sp. nov. (Gastrotricha: Chaetonotida: Chaetonotidae) from a palm house in Vienna (Austria). *Ann. Zool.* 69, 447–475. doi: 10.3161/00034541ANZ2019.69.2.011.

Medlin, L., Elwood, H. J., Stickel, S., and Sogin, M. L. (1988). The characterization of enzymatically amplified eukaryotic 16S-like rRNA-coding regions. *Gene* 71, 491–499. doi: 10.1016/0378-1119(88)90066-2

Miao, M., Warren, A., Song, W., Wang, S., Shang, H., and Chen, Z. (2008). Analysis of the internal transcribed spacer 2 (ITS2) region of scuticociliates and related taxa (Ciliophora, Oligohymenophorea) to infer their evolution and phylogeny. *Protist* 159, 519–533. doi: 10.1016/j.protis.2008.05.002

Obert, T., and Vďačný, P. (2019). Integrative taxonomy of five astome ciliates (Ciliophora, Astomatia) isolated from earthworms in Central Europe. *Eur. J. Taxon*. 559, 1–37. doi: 10.5852/ejt.2019.559

Obert, T., Rurik, I., and Vďačný, P. (2021). Diversity and eco-evolutionary associations of endosymbiotic astome ciliates with their lumbricid earthworm hosts. *Front. Microbiol.* 12, e689987. doi: 10.3389/fmicb.2021.689987

Park, M. H., Jung, J. H., Jo, E., Park, K. M., Baek, Y. S., Kim, S. J., and Min, G. S. (2019). Utility of mitochondrial CO1 sequences for species discrimination of Spirotrichea ciliates (Protozoa, Ciliophora). *Mitochon*. *DNA Part A* 30, 148–155. doi: 10.1080/24701394.2018.1464563

Pawlowski, J. (2000). Introduction to the molecular systematics of foraminifera. *Micropaleontology* 46 (Suppl. 1), 1–12.

Pérez-Losada, M., Ricoy, M., Marshall, J. C., and Domínguez, J. (2009). Phylogenetic assessment of the earthworm *Aporrectodea caliginosa* species complex (Oligochaeta: Lumbricidae) based on mitochondrial and nuclear DNA sequences. *Mol. Phylogenet. Evol.* 52, 293–302. doi: 10.1016/j.ympev.2009.04.003

Rataj, M., and Vďačný, P. (2020). Multi-gene phylogeny of *Tetrahymena* refreshed with three new histophagous species invading freshwater planarians. *Parasitol. Res*. 119, 1523–1545. doi: 10.1007/s00436-020-06628-0

Vďačný, P., Bourland, W. A., Orsi, W., Epstein, S. S., and Foissner, W. (2011). Phylogeny and classification of the Litostomatea (Protista, Ciliophora), with emphasis on free-living taxa and the 18S rRNA gene. *Mol. Phylogenet. Evol.* 59, 510–522. doi: 10.1016/j.ympev.2011.02.016
